# Transcription factor NKX2–1 drives serine and glycine synthesis addiction in cancer

**DOI:** 10.1038/s41416-023-02216-y

**Published:** 2023-03-17

**Authors:** Elien Heylen, Paulien Verstraete, Linde Van Aerschot, Shauni L. Geeraerts, Tom Venken, Kalina Timcheva, David Nittner, Jelle Verbeeck, Jonathan Royaert, Marion Gijbels, Anne Uyttebroeck, Heidi Segers, Diether Lambrechts, Jan Cools, Kim De Keersmaecker, Kim R. Kampen

**Affiliations:** 1 Laboratory for Disease Mechanisms in Cancer, Department of Oncology, KU Leuven, Leuven, Belgium; 2 Leuven Cancer Institute (LKI), Leuven, Belgium; 3 Laboratory for Translational Genetics, Department of Human Genetics, KU Leuven, Leuven, Belgium; 4 Center for Cancer Biology, VIB, Leuven, Belgium; 5 Histopathology Expertise Center, VIB-KU Leuven Center for Cancer Biology, VIB, Leuven, Belgium; 6 Department of Oncology, KU Leuven, Leuven, Belgium; 7 Department of Pathology, GROW School for Oncology and Reproduction, Maastricht, The Netherlands; 8 Department of Medical Biochemistry, Experimental Vascular Biology, Amsterdam Cardiovascular Sciences, Amsterdam Infection and Immunity, Amsterdam UMC, Amsterdam, The Netherlands; 9 Paediatric Haematology and Oncology, University Hospitals Leuven, Leuven, Belgium; 10 Department of Oncology, Paediatric Oncology, KU Leuven, Leuven, Belgium; 11 Laboratory of Molecular Biology of Leukemia, Department of Human Genetics, KU Leuven, Leuven, Belgium; 12 Maastricht University Medical Centre, Department of Radiation Oncology (MAASTRO), GROW School for Oncology and Reproduction, Maastricht, The Netherlands

**Keywords:** Cancer metabolism, Targeted therapies, Acute lymphocytic leukaemia, Non-small-cell lung cancer, Oncogenes

## Abstract

**Background:**

One-third of cancers activate endogenous synthesis of serine/glycine, and can become addicted to this pathway to sustain proliferation and survival. Mechanisms driving this metabolic rewiring remain largely unknown.

**Methods:**

NKX2–1 overexpressing and NKX2–1 knockdown/knockout T-cell leukaemia and lung cancer cell line models were established to study metabolic rewiring using ChIP-qPCR, immunoblotting, mass spectrometry, and proliferation and invasion assays. Findings and therapeutic relevance were validated in mouse models and confirmed in patient datasets.

**Results:**

Exploring T-cell leukaemia, lung cancer and neuroendocrine prostate cancer patient datasets highlighted the transcription factor NKX2–1 as putative driver of serine/glycine metabolism. We demonstrate that transcription factor NKX2–1 binds and transcriptionally upregulates serine/glycine synthesis enzyme genes, enabling NKX2–1 expressing cells to proliferate and invade in serine/glycine-depleted conditions. NKX2–1 driven serine/glycine synthesis generates nucleotides and redox molecules, and is associated with an altered cellular lipidome and methylome. Accordingly, NKX2–1 tumour-bearing mice display enhanced tumour aggressiveness associated with systemic metabolic rewiring. Therapeutically, NKX2–1-expressing cancer cells are more sensitive to serine/glycine conversion inhibition by repurposed anti-depressant sertraline, and to etoposide chemotherapy.

**Conclusion:**

Collectively, we identify NKX2–1 as a novel transcriptional regulator of serine/glycine synthesis addiction across cancers, revealing a therapeutic vulnerability of NKX2–1-driven cancers.

**Figure:**
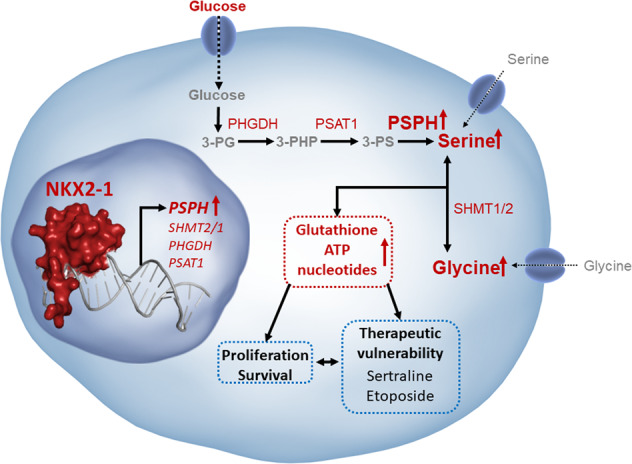
Transcription factor NKX2–1 fuels cancer cell proliferation and survival by hyperactivating serine/glycine synthesis, highlighting this pathway as a novel therapeutic target in NKX2–1-positive cancers.

## Introduction

**Fig. 1:**
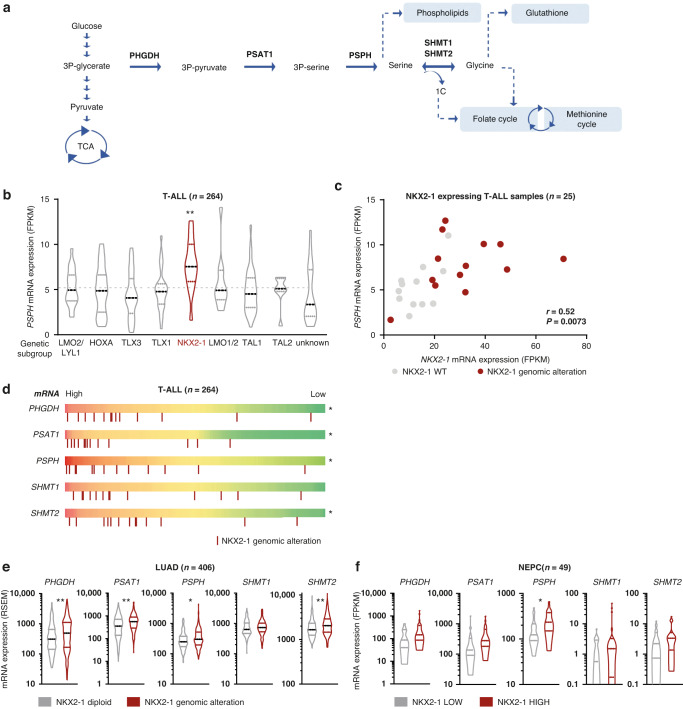
NKX2–1 overexpression is associated with elevated mRNA expression of serine and glycine synthesis enzymes in T-ALL, LUAD and NEPC. **a** Schematic overview of the serine/glycine synthesis pathway. **b** *PSPH* mRNA expression levels according to genetic subgroup in 264 T-ALL patients [15] (*P* = 0.0014; Dunnett’s multiple comparisons test). **c** *PSPH* mRNA expression levels correlated to *NKX2–1* mRNA expression levels in 25 NKX2–1 expressing T-ALL samples. Red dots represent NKX2–1 genetically altered samples (Pearson correlation). **d** The same 264 T-ALL patient samples as in (**b**) ranked according to mRNA expression levels of the according serine/glycine synthesis enzymes (red = high expression of the indicated enzyme; green =  low expression of the indicated enzyme). Red bars below the mRNA expression scale bar indicate patient samples with NKX2–1 genomic alterations (Fisher’s exact test). **e** Serine/glycine synthesis enzyme mRNA expression levels in *NKX2–1* diploid versus *NKX2–1* amplified LUAD patients (*n* = 406; Welch’s *t* test). **f** Serine/glycine synthesis enzyme mRNA expression levels in NEPC tumours with high *NKX2–1* expression (50% highest expressers) versus low *NKX2–1* expression (50% lowest expressers) (*n* = 49; unpaired *t* test). All violin plots show the median and quartiles. Statistical analysis *P value < 0.05, **P value < 0.01, ***P value < 0.001.

During tumorigenesis, cancer cells reprogramme their energy metabolism to facilitate growth and enhance cell survival [1]. For example, while the majority of normal cells take up serine and glycine from their environment, a growing list of cancer subtypes activate intracellular serine/glycine synthesis and become addicted to this to maintain themselves and their surrounding niches [2–4]. Serine/glycine synthesis branches from glycolysis via the glycolytic intermediate 3-phospho-glycerate (3-PG) (Fig. 1a). 3-PG can be converted into serine via three enzymatic reactions catalysed by 3-phosphoglycerate dehydrogenase (PHGDH), phosphoserine aminotransferase (PSAT1) and phosphoserine phosphatase (PSPH), respectively. Next, serine can be reversibly converted into glycine by serine hydroxymethyltransferase (SHMT) 1, residing in the cytosol, or SHMT2 in the mitochondria. This enzyme-driven conversion can be inhibited by the repurposed anti-depressant sertraline [5]. Serine and glycine are required for important cellular biosynthetic processes such as the production of phospholipids and redox homeostasis molecules. Moreover, serine supplies one-carbon units to the folate cycle, which fuels de novo purine synthesis and facilitates methylation of homocysteine, an essential process for DNA and histone methylation [2, 4].

Previously, several oncogenic genomic alterations that promote serine synthesis have been identified. In melanoma and breast cancer, PHGDH amplifications were described [6, 7]. MYC overexpression in B-cell lymphoma transcriptionally increases serine synthesis enzyme levels [8]. In addition, activating KRAS mutations in non-small cell lung cancer (NSCLC), pancreatic and intestinal cancers enhance serine synthesis by increasing transcription of NRF2, a master regulator that promotes serine/glycine synthesis enzyme gene expression through ATF4 activation [9–11]. This enables KRAS mutant tumours to grow in the absence of available serine/glycine for uptake [9]. Recently, we showed that the ribosomal RPL10 R98S mutation enhances serine/glycine synthesis in T-cell acute lymphoblastic leukaemia (T-ALL) by elevating both *PSPH* transcription and translation [12].

T-ALL is an aggressive haematologic cancer caused by the accumulation of somatic mutations in developing T cells [13]. T-ALL patients are classified into subgroups based on their transcriptional profile and the mutually exclusive ectopic expression of transcription factors, such as LMO1/2, HOXA, TLX1/3, NKX2–1 or TAL1/2. Other hallmarks of T-ALL include the inactivation of the tumour suppressors CDKN2A/CDKN2B, hyperactivation of the NOTCH1 cascade and mutations in PHF6, PTEN and the JAK-STAT signalling pathway [13]. Interestingly, we noted that expression levels of serine/glycine synthesis enzymes, particularly of *PSPH*, are elevated by at least fourfold in 80% of all T-ALL samples, whereas the RPL10 R98S mutation is only present in 8% of the samples [12, 14, 15]. Moreover, *PSPH* knockdown in three RPL10 R98S negative T-ALL cell lines impaired leukaemic cell proliferation and reduced in vivo expansion potential of the T-ALL cells in patient-derived xenograft (PDX) mouse models, demonstrating that also RPL10 R98S negative T-ALL samples depend on high PSPH expression levels for their expansion capacity [12]. Accordingly, SHMT inhibition has recently been identified as a major therapeutic vulnerability in T-ALL [16, 17]. Altogether, these data support that other causes of serine/glycine synthesis addiction besides the RPL10 R98S mutation in T-ALL remain to be discovered.

In this work, we identify NKX2–1 as a transcription factor driving the overexpression of serine/glycine synthesis enzymes and causing cell addiction to this pathway in cancer. Genetic alterations in NKX2–1 are common in cancer. Around 5% of T-ALL patients display chromosomal rearrangements or amplifications driving expression of NKX2–1, a homeodomain transcription factor that is not expressed during normal T-cell development [15, 18]. Also, 12% of lung adenocarcinoma (LUAD) samples display gene amplifications in NKX2–1, driving its overexpression [19, 20]. In addition, 16% of the neuroendocrine prostate cancer (NEPC) cases overexpress *NKX2–1* and this overexpression has been identified as a driver of prostate adenocarcinoma to NEPC reprogramming [21, 22].

Here, we demonstrate that NKX2–1 overexpression results in extensive metabolic rewiring towards serine/glycine synthesis, enabling these cells to proliferate upon limited serine/glycine availability. Despite the improved outcomes of T-ALL, LUAD and NEPC patients, treatment-related toxicity and therapy resistance remain major problems [23–25]. In this manuscript, we highlight altered serine/glycine metabolism as key target in NKX2–1-driven cancers to improve current treatment regimens. Importantly, we show that PSPH is essential for the proliferation of NKX2–1 expressing cancer cells, and we uncover that NKX2–1 overexpressing cells are sensitised to metabolic drugs, including the dual SHMT1/2 inhibitor sertraline and the topoisomerase II inhibitor etoposide. The latter is of direct clinical relevance and translates into better survival for NKX2–1 overexpressing cancer patients receiving etoposide-based treatments and emphasizes novel future treatment perspectives for NKX2–1-positive cancers with more specificity and less toxicity using sertraline.

## Results

### NKX2–1-positive tumours express higher mRNA levels of serine and glycine synthesis enzyme genes

We previously showed that normal thymocytes and bone marrow cells display low *PSPH* expression levels, whereas 95% of analysed T-ALL cell lines and 80% of T-ALL patients express *PSPH* mRNA levels that are at least fourfold higher [12, 14]. In order to identify the genetic T-ALL subgroups associated with the highest *PSPH* expression, we analysed RNA and exome sequencing data from 264 T-ALL samples [15]. The *NKX2–1* subgroup presented with the significantly highest *PSPH* mRNA expression compared to all other T-ALL patient subgroups (Fig. 1b) [15]. In addition, we observed a significant correlation between *NKX2–1* and *PSPH* expression in all *NKX2–1* expressing T-ALL samples (Fig. 1c). NKX2–1 is a homeodomain transcription factor with a role in the development of the thyroid, lungs and part of the brain [26]. Although it is not expressed during normal T-cell development, 5% of T-ALL samples express high *NKX2–1* levels due to chromosomal translocations or genomic amplifications (referred to as *NKX2–1* altered samples below) [15, 18]. Strikingly, when ranking T-ALL samples according to mRNA expression level of the different serine/glycine synthesis enzymes (*PHGDH*, *PSAT1*, *PSPH*, *SHMT1* and *SHMT2*), *NKX2–1* altered samples clustered on the side of the samples with the highest expression of all serine/glycine pathway enzymes (Fig. 1d). Conformingly, besides *PSPH*, the *NKX2–1* subgroup also showed significantly highest *PHGDH*, *PSAT1* and *SHMT2* expression compared to all other patient samples (Supplementary Fig. 1).

Because *NKX2–1* copy number gains also have been identified in LUAD and NEPC, we investigated whether NKX2–1 associated elevation of serine/glycine synthesis enzyme expression is uniformly observed across all cancer types. In LUAD, *NKX2–1* copy number gain was associated with up to 40% increased expression levels of *PHGDH*, *PSAT1*, *PSPH* and *SHMT2* as compared to samples with a diploid *NKX2–1* status (Fig. 1e). NEPC is a rare tumour, and only 49 samples could be analysed, limiting statistical power. Nevertheless, *PSPH* mRNA expression was 1.7-fold elevated in the 50% of NEPC tumours with the highest *NKX2–1* expression versus the 50% with the lowest *NKX2–1* expression (Fig. 1f). These results show that *NKX2–1* overexpression is associated with elevated mRNA levels of serine/glycine synthesis enzymes in T-ALL, LUAD and NEPC.

### NKX2–1 is a direct transcriptional inducer of serine/glycine synthesis enzymes

**Fig. 2:**
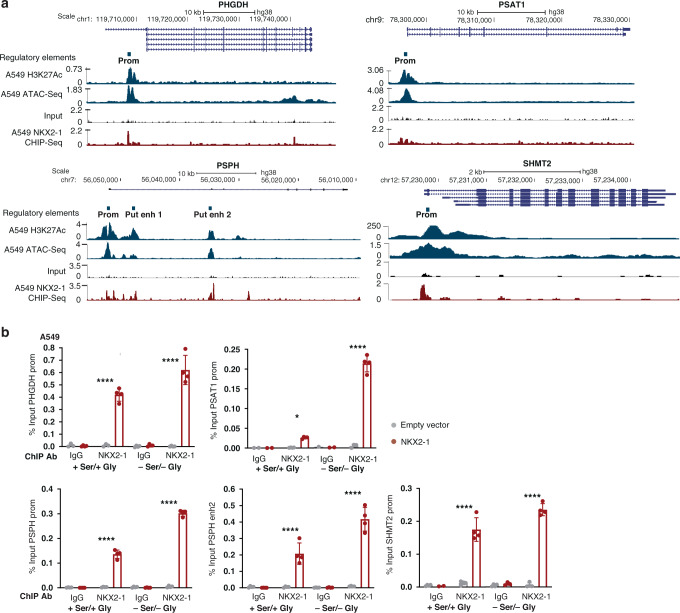
NKX2–1 directly binds regulatory sequences in serine/glycine synthesis enzyme encoding genes. **a** Genomic loci of the *PHGDH*, *PSAT1*, *PSPH* and *SHMT2* genes downloaded from the UCSC genome browser (hg38). H3K27 acetylation and ATAC-Seq is shown as a marker of open chromatin. A549 DNA input and NKX2–1 ChIP-Seq peaks obtained from this cell line were downloaded from the Cistrome data browser. Signals are plotted as normalised read counts. **b** NKX2–1 ChIP-qPCR results for the *PHGDH* promoter, *PSAT1* promoter, *PSPH* promoter and putative enhancer 2 region and *SHMT2* promoter obtained in in A549 cells cultured with or without serine and glycine (*n* = 4 technical replicates). Data are represented as mean ± standard deviation. Individual dots represent independent observations. Statistical analysis *P value < 0.05, **P value < 0.01, ***P value < 0.001, ****P value < 0.0001. *P* values were calculated using a two-tailed Student’s *t* test.

Since NKX2–1 is a transcription factor, we hypothesised that NKX2–1 may act as a direct transcriptional activator of serine/glycine synthesis enzyme gene expression. To test this hypothesis, we consulted available ChIP-Seq data of NKX2–1 overexpressing A549 LUAD cells [27]. These data revealed NKX2–1 binding at the *PSPH*, the *PHGDH* and the *SHMT2* promoter and at two putative *PSPH* enhancer regions that correspond to open chromatin regions as indicated by H3K27 acetylation and ATAC-seq signals (Fig. 2a) [28]. A smaller NKX2–1 binding peak was found in the *PSAT1* promoter (Fig. 2a). Furthermore, by using Cluster-Buster (cBust) [29], we identified the occurrence of NKX2 motif clusters in the sequence of the *PHGDH* and *PSPH* promoter and *PSPH* enhancer regions (Supplementary Fig. 2a, b).

To confirm these findings, we transduced DND41 T-ALL and A549 LUAD cancer cell lines with an NKX2–1 overexpression vector. NKX2–1 ChIP-qPCR on these cells confirmed specific NKX2–1 binding to the *PHGDH*, *PSAT1*, *PSPH* and *SHMT2* promoter and *PSPH* putative enhancer region 2 (Fig. 2b and Supplementary Fig. 2c,d). Furthermore, we observed that the binding of NKX2–1 to the regulatory regions of serine/glycine pathway enzymes increased up to fourfold in response to serine and glycine depletion (Fig. 2b).

**Fig. 3:**
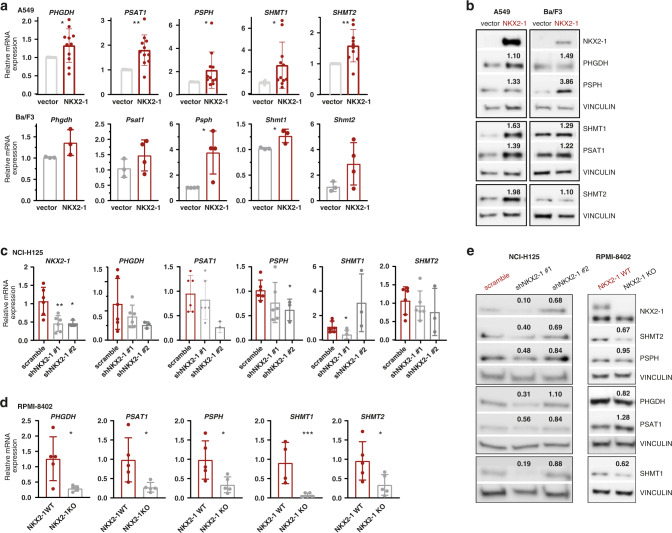
NKX2–1 expression is associated with increased mRNA and protein levels of serine/glycine synthesis enzymes. **a** Relative mRNA expression levels of *PHGDH, PSAT1, PSPH, SHMT1* and *SHMT2* in empty vector control versus *NKX2–1* overexpressing A549 and Ba/F3 cells (*n* ≥ 3 biological replicates). **b** Immunoblot analysis of NKX2–1, PHGDH, PSAT1, PSPH, SHMT1 and SHMT2 expression in empty vector control versus *NKX2–1* overexpressing A549 and Ba/F3 cells. The mean fold increase upon *NKX2–1* overexpression is indicated on the immunoblot. **c** Relative mRNA expression levels of *NKX2–1, PHGDH, PSAT1, PSPH, SHMT1* and *SHMT2* in scramble control versus *NKX2–1* knockdown NCI-H125 cells (*n* ≥ 4 biological replicates). Only samples with at least 20% *NKX2–1* knockdown on RNA level were considered. **d** Relative mRNA expression levels of *PHGDH, PSAT1, PSPH, SHMT1* and *SHMT2* in *NKX2–1* WT versus CRISPR-Cas9 *NKX2–1* KO RPMI-8402 cells (*n* = 5 biological replicates). **e** Immunoblot analysis of NKX2–1, PHGDH, PSAT1, PSPH, SHMT1 and SHMT2 expression in scramble control versus *NKX2–1* knockdown NCI-H125 and in *NKX2–1* WT versus CRISPR-Cas9 *NKX2–1* KO RPMI-8402 cells. The mean fold decrease upon *NKX2–1* knockdown/knockout is indicated on the immunoblot. Data are represented as mean ± standard deviation. Individual dots represent independent observations. Statistical analysis *P value < 0.05, **P value < 0.01, ***P value < 0.001, ****P value < 0.0001. *P* values were calculated using a two-tailed Student’s *t* test. All western blots shown are representative examples. All cells were cultured in medium without serine/glycine for 4 h or 48 h (RPMI-8402 cells for immunoblotting experiments) prior to pellet collection.

Since serine/glycine poor cell culture media better reflect the intra-tumoral state than standard cell culture media and NKX2–1 binding to the regulatory regions of serine/glycine pathway enzymes increased in response to serine/glycine depletion, we depleted the cell culture medium from serine and glycine for all following experiments. In NKX2–1 overexpressing A549 cells, mRNA and protein expression of all serine/glycine synthesis enzymes was increased up to 2.5-fold as compared to vector transduced control cells (Fig. 3a, b and Supplementary Fig. 3a). In NKX2–1 overexpressing Ba/F3 cells, a mouse lymphoid pro-B-cell line, mRNA and protein expression of one or more serine/glycine synthesis enzymes was significantly increased 1.2- to 3.8-fold (Fig. 3a, b and Supplementary Fig. 3b).

To test our hypothesis in a reverse way, we analysed the impact of shRNA-mediated *NKX2–1* knockdown on serine/glycine synthesis enzyme expression in LUAD cell line NCI-H125 that endogenously expresses *NKX2–1*. *NKX2–1* knockdown in NCI-H125 cells resulted in a 20–80% decrease of serine/glycine synthesis enzyme mRNA and protein expression levels (Fig. 3c, e and Supplementary Fig. 3c). Of note, shNKX2-1 #2 was less effective in decreasing NKX2–1 protein levels, thereby also resulting in a less pronounced effect on serine/glycine synthesis enzyme protein expression levels compared to shNKX2-1 #1. In addition, we knocked out *NKX2–1* via CRISPR-Cas9 technology in T-ALL cell line RPMI-8402 that endogenously expresses *NKX2–1*. *NKX2–1* knockout in RPMI-8402 cells resulted in an 80% reduction of serine/glycine synthesis enzyme RNA expression levels, although without a significant decrease in protein expression levels (Fig. 3d, e and Supplementary Fig. 3d). RPMI-8402 cells not only express *NKX2–1*, but also NKL homeobox genes *NKX2-2* and *NKX3-1*, which share similar downstream effects [30, 31]. Therefore, these limited effects of *NKX2–1* knockout may be attributed to some extent to functional redundancy of these transcription factors. Altogether, our observations support that NKX2–1 is a direct regulator of serine/glycine synthesis enzyme expression in various cellular contexts.

### NKX2–1 expression induces de novo serine/glycine synthesis to control ATP supply, nucleotide metabolism and redox homeostasis

**Fig. 4:**
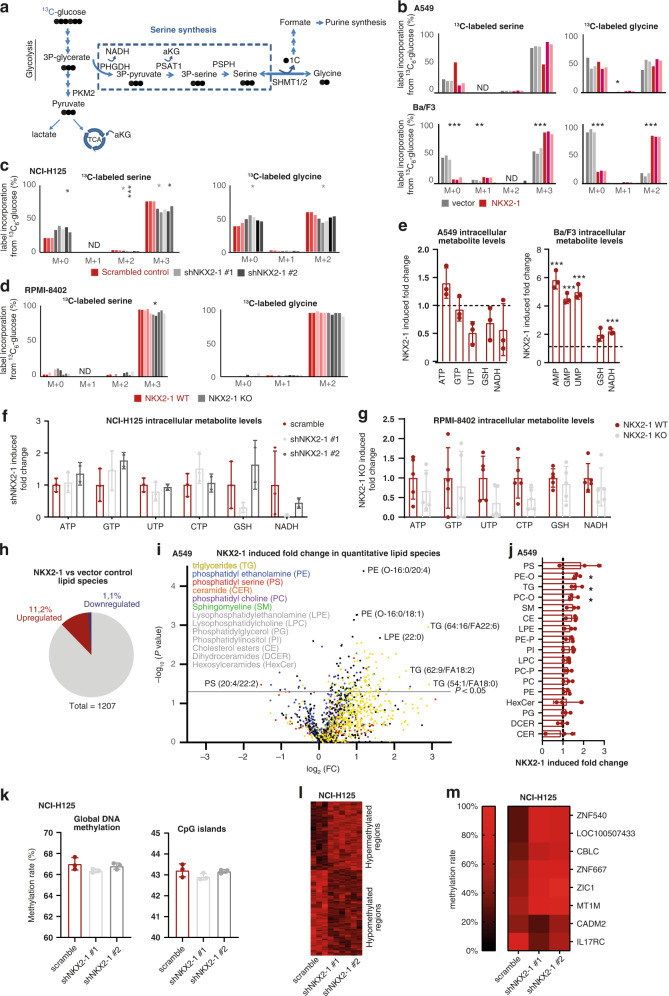
NKX2–1 expression induces serine/glycine synthesis and downstream metabolic pathways and alters the cellular lipidome and methylome. **a** Schematic overview of ^13^C_6_-glucose label distribution into the serine/glycine synthesis pathway. **b**–**d** Metabolic tracer analysis using ^13^C_6_-glucose, measuring labelled serine/glycine in empty vector control versus *NKX2–1* overexpressing A549 cells (*n* = 3) and in Ba/F3 cells (*n* = 3) (**b**) and in scramble control versus *NKX2–1* knockdown NCI-H125 cells (*n* = 3 for scramble and shNKX2-1 #1; *n* = 2 for shNKX2-1 #2; statistics per hairpin are displayed in the corresponding hairpin colour)(**c**) and *NKX2–1* WT versus CRISPR-Cas9 *NKX2–1* KO RPMI-8402 cells (*n* = 5) (**d**). **e**–**g** Total intracellular metabolite levels in empty vector control versus *NKX2–1* overexpressing A549 cells (*n* = 3) and in Ba/F3 cells (*n* = 3) (**e**) and in scramble control versus *NKX2–1* knockdown NCI-H125 cells (*n* = 3 for scramble and shNKX2-1 #1; *n* = 2 for shNKX2-1 #2) (**f**) and *NKX2–1* WT versus CRISPR-Cas9 *NKX2–1* KO RPMI-8402 cells (*n* = 5) (**g**). **h** Pie chart reporting the fraction of detected lipids that are significantly up- or downregulated in *NKX2–1* overexpressing A549 cells (*n* = 3). **i** Volcano plot of the quantitative lipidomics data comparing empty vector control and *NKX2–1* overexpressing A549 samples (*n* = 3). The line indicates the cut-off for significance (*P* < 0.05; *t* test). The colours of the dots represent the lipid class to which each individual species belongs. **j** *NKX2–1* overexpression induced fold change in intracellular lipid classes in empty vector control versus NKX2–1 overexpressing A549 cells (*n* = 3). **k** Global DNA methylation rate (Bismark) and DNA methylation rate at CpG islands in scramble control versus *NKX2–1* knockdown NCI-H125 cells (*n* = 3). **l** Heatmap representing methylation rate of differentially methylated regions (250 bp) in scramble control versus *NKX2–1* knockdown NCI-H125 cells (*n* = 3) (*q*-value < 0.01, methylation difference >0.2). Only differentially methylated regions without missing CpG counts across all samples are shown for clarity. **m** Heatmap representing methylation rate of genes connected to differentially methylated regions in scramble control versus *NKX2–1* knockdown NCI-H125 cells (*n* = 3). Data are represented as mean ± standard deviation. Individual dots represent independent observations. Statistical analysis *P value < 0.05, **P value < 0.01, ***P value < 0.001, ****P value < 0.0001. *P* values were calculated using a two-tailed Student’s *t* test. All cells were cultured in medium without serine/glycine for 4 h prior to pellet collection. ND non-determined, -O 1-alkyl,2-acyl, -P 1-alkenyl,2-acyl.

Next, we explored whether the NKX2–1-driven increased expression of serine/glycine synthesis enzymes causes metabolic rewiring of cancer cells. We carried out mass spectrometry-based ^13^C_6_-glucose tracing experiments, in which synthesis of serine and glycine can be tracked by following carbon-13 incorporation. Serine and glycine synthesised from glucose will display mass shifts of 3 and 2 units (M + 3 serine and M + 2 glycine), unlabelled (M + 0) serine and glycine will originate from cellular uptake, and interconversion between serine and glycine will give rise to partially labelled serine (M + 1 and M + 2) (Fig. 4a). As intracellular and extracellular serine levels of *NKX2–1* overexpressing A549 cells were significantly lower compared to control cells, we were not able to observe a significant increase in M + 3 serine and M + 2 glycine in these cells, indicating that *NKX2–1* expressing cells use all produced serine for intracellular downstream purposes (Fig. 4b and Supplementary Fig. 4a). *NKX2–1* overexpression enabled Ba/F3 cells to synthesise serine and glycine from glucose, presenting a 1.6-fold increased M + 3 serine and a 5-fold increased M + 2 glycine as compared to vector control cells (Fig. 4b). Conversely, the de novo serine/glycine synthesis potential was significantly decreased by >20%, upon *NKX2–1* knockdown in NCI-H125 cells (Fig. 4c). The *NKX2–1* knockdown induced effects on de novo serine synthesis were NKX2–1 dose dependent, as more profound effects were observed in shNKX2-1 #1 than in shNKX2-1 #2 (less effective hairpin) knockdown cells. Also in RPMI-8402 cells, *NKX2–1* knockout slightly reduced the level of M + 3 serine, but not of M + 2 glycine (Fig. 4d). Of interest, whereas extracellular levels of serine were unchanged between NKX2–1 WT and KO RPMI-8402 cells, extracellular levels of all other amino acids, and most strikingly alanine, were drastically decreased in *NKX2–1* KO RPMI-8402 cells compared to WT (Supplementary Fig. 5a). This suggests that *NKX2–1* KO cells compensate the loss of serine/glycine synthesis enzymes via activation of an alternative mechanism. As such, alanine can be converted into serine via serine-pyruvate transaminase [32]. In this reaction, the carbons of serine would be originating from 3-hydroxypyruvate and will also contain three labelled carbons, which can explain why we only see a slight reduction in M + 3 serine and M + 2 glycine upon *NKX2–1* knockout in these cells. In line with this finding, *NKX2–1* overexpressing A549 cells also presented elevated labelled alanine levels, indicating an increased de novo production (Supplementary Fig. 5b).

In addition to the ^13^C_6_-glucose tracing experiments, we measured total cellular metabolite levels. No increases in serine or glycine levels were observed in NKX2–1 expressing cells (Supplementary Fig. 4a–f). This suggests that all produced serine/glycine in NKX2–1 expressing cells is utilised to generate downstream metabolites. As the serine synthesis pathway is interconnected with diverse metabolic processes (Fig. 1a) [2–4], we observed extensive NKX2–1-driven oncogenic metabolic rewiring. Of note, the most elevated metabolites in *NKX2–1* overexpressing versus control and (scramble) control versus *NKX2–1* knockdown/knockout cells were related to ATP synthesis, nucleotide metabolism and redox homeostasis (Fig. 4e–g and Supplementary Figs. 6 and 7).

Purine synthesis is tightly connected to the serine/glycine synthesis pathway since it requires the direct contribution of carbons and nitrogens from glycine and formate, which both depend on SHMT1/2 [2, 4]. Correspondingly, the intracellular purine levels were increased up to 1.5-fold in *NKX2–1* overexpressing A549 cells and up to sixfold in *NKX2–1* overexpressing Ba/F3 cells compared to control (Fig. 4e and Supplementary Fig. 6a). Also, in *NKX2–1* KO RPMI-8402 cells, intracellular levels of the purines ATP and GTP were decreased by up to 40% (Fig. 4g and Supplementary Fig. 6c). Synthesis of purines from serine/glycine can also be tracked by ^13^C_6_-glucose tracing as ^13^C_6_-glucose-derived carbon is incorporated into ATP through the production of ribose (M + 5) and through serine contribution to the purine ring via 10-formyl-THF (M + 1 or M + 2) and glycine (M + 2). Higher-order labelled species of ATP (M + 6–9) from ^13^C_6_-glucose thus directly originate from de novo produced serine and glycine. As such, we observed a significant threefold decrease in M + 5 ATP and increases in higher-order labelled species of ATP (M + 6–9) in *NKX2–1* overexpressing versus control Ba/F3 cells and a twofold increase in M + 5 ATP and decreases in M + 6–9 ATP upon *NKX2–1* knockdown with shNKX2-1 #1 in NCI-H125 cells (Supplementary Fig. 7a, b).

Since increased anti-oxidative capacity enhances tumorigenesis in various cancer settings, we next focused on cellular redox homeostasis controlled by glutathione (GSH) [33]. As GSH is composed of cysteine, glycine and glutamate, its synthesis heavily relies on the serine/glycine synthesis pathway [2, 4]. Aside from its conversion into glycine, serine also contributes to cysteine production through the trans-sulfuration pathway [2]. Strikingly, upon *NKX2–1* overexpression, intracellular GSH levels increased twofold in Ba/F3 cells (Fig. 4e). In contrast, upon *NKX2–1* knockout, intracellular GSH levels decreased in RPMI-8402 cells (Fig. 4g). Furthermore, ^13^C-glucose-derived carbons that are incorporated into GSH are mainly derived from glycine (M + 2) and glutamate (M + 2). As such, in A549 and Ba/F3 cells, we observed an NKX2–1-driven increase in M + 4 GSH (Supplementary Fig. 7d). In line with this, *NKX2–1* overexpressing A549 cells displayed a 50% reduction in intracellular reactive oxygen species (ROS) levels compared to control A549 cells (Supplementary Fig. 8a). In contrast, *NKX2–1* knockdown in NCI-H125 cells and *NKX2–1* knockout in RPMI-8402 cells increased intracellular ROS levels (Supplementary Fig. 8b, c). Changes in ROS were not detected in *NKX2–1* overexpressing Ba/F3 cells, which are more nucleotide synthesis oriented (Supplementary Fig. 8a). Confirmingly, *NKX2–1* overexpressing A549 cells were more resistant to oxidative stress induction by hydrogen peroxide (H_2_O_2_) compared to control cells, which was again unchanged in Ba/F3 cells (Supplementary Fig. 8d, e). Taken together, these observations highlight that NKX2–1-driven de novo serine/glycine synthesis output depends on the cellular needs and context. NKX2–1 overexpression boosts the antioxidant capacity of LUAD cells, whereas T-ALL cells preferably use NKX2–1 expression to increase nucleotide synthesis.

### NKX2–1 overexpression alters the lipidome and methylome of A549 cells

Serine is important to sustain lipid synthesis. In this regard, serine is directly used to synthesise phospholipids phosphatidylserine (PS), phosphatidylethanolamine (PE) and phosphatidylcholine [2]. In addition, together with palmitoyl-CoA, serine serves as building block of sphingolipids including ceramides and sphingomyelins and in conditions of limited serine availability, also l-alanine may serve as a substrate for serine palmitoyl transferase reactions [2, 34]. Yet, the effect of serine synthesis upregulation on lipid metabolism in cancer is relatively unexplored. Therefore, we assessed the impact of NKX2–1 overexpression on lipid metabolism. Strikingly, 11.2% of the 1207 detected lipid species were significantly upregulated in A549 cells upon NKX2–1 overexpression, opposed to only 1.1% that were downregulated (Fig. 4h, i). The lipid classes with the strongest upregulation upon NKX2–1 overexpression were PS, ether-linked PE, and surprisingly, triglycerides (Fig. 4j). Notably, ceramide levels were not affected, whereas we observed an increased abundance of sphingomyelins, which can be derived from ceramide (*P* = 0.06).

Furthermore, serine/glycine synthesis can regulate cellular epigenetics by for example fuelling the methionine salvage pathway, which contributes to the production of S-adenosyl methionine (SAM), the substrate for methylation of DNA [35, 36]. To characterise the impact of NKX2–1 overexpression on DNA methylation, we performed low-coverage whole-genome sequencing after enzymatic oxidation and deamination of DNA, thereby protecting methylated cytosines and converting unmethylated cytosines into uracils. This analysis demonstrated a trend towards decreased global DNA and CpG island methylation in *NKX2–1* knockdown NCI-H125 cells compared to scramble control cells (Fig. 4k). Comparison of methylation levels at 250 bp regions revealed 168 hypermethylated and 206 hypomethylated regions in *NKX2–1* knockdown cells (*q*-value <0.01, methylation difference >0.2) (Fig. 4l). Finally, annotation of the differentially methylated regions identified two hypomethylated and six hypermethylated gene promotors in *NKX2–1* knockdown cells compared to scramble control (Fig. 4m). In conclusion, NKX2–1 overexpression alters both the lipidome and epigenetic landscape of cancer cells.

### NKX2–1-driven metabolic independence supports cancer cell proliferation and invasion

**Fig. 5:**
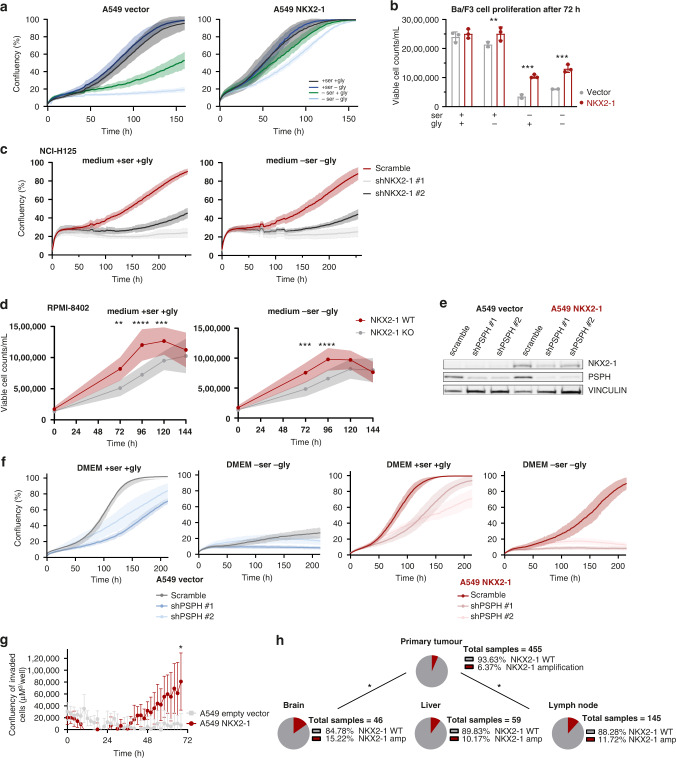
NKX2–1 driven metabolic rewiring supports cancer cell proliferation and invasion. **a** Cell confluency over time of empty vector control versus *NKX2–1* overexpressing A549 cells cultured with/without serine and/or glycine. A representative experiment (*n* = 3 biological replicates) with six technical replicates is shown. **b** Viable Ba/F3 cell counts/mL after 72 h of culturing in RPMI-1640 with/without serine and/or glycine. Viability was determined by annexin V-PE/Zombie aqua flow cytometry (*n* = 3). **c** Cell confluency over time of scramble control versus *NKX2–1* knockdown NCI-H125 cells cultured with or without serine and glycine. A representative experiment (*n* = 3 biological replicates) with six technical replicates is shown. **d** Viable *NKX2–1* WT vs CRISPR-Cas9 *NKX2–1* KO RPMI-8402 cell counts/mL over time cultured in RPMI-1640 with or without serine/glycine. Viable cell counts were determined based on scatter measured on a Guava flow cytometer (*n* = 9). **e** Immunoblot analysis of empty vector control and *NKX2–1* overexpressing A549 cells after transduction with scramble control or *PSPH* knockdown vector. Immunoblots are representative examples of at least three replicates. **f** Cell confluency over time of scramble control versus *PSPH* knockdown empty vector and *NKX2–1* overexpressing A549 cells cultured with or without serine and glycine. A representative experiment with six technical replicates is shown. **g** Cell invasion over time of empty vector control and *NKX2–1* overexpressing A549 cells towards an FCS gradient quantified by measuring the area of invaded cells on the bottom plate of an Incucyte clearview plate (*n* = 4). **h** *NKX2–1* mutational state according to site of tissue sampling in non-small lung cancer patients [39]. *P* values were calculated using a Fisher’s exact test. Data are represented as mean ± standard deviation. Individual dots represent independent observations. Statistical analysis *P value < 0.05, **P value < 0.01, ***P value < 0.001, ****P value < 0.0001. *P* values were calculated using a two-tailed Student’s *t* test unless otherwise indicated.

In the presence of serine, *NKX2–1* overexpressing A549 and Ba/F3 cells showed a slight proliferation benefit compared to control cells. However, as most cell lines require serine supply in the culture medium for their proliferation [2–4], when cultured in medium lacking serine, control cells were unable to proliferate, as opposed to *NKX2–1* overexpressing cells, for which proliferation continued (Fig. 5a, b). Notably, *NKX2–1* knockdown or knockout strongly impaired the proliferation of NCI-H125 and RPMI-8402 cells compared to scramble control, both in medium with and without serine and glycine (Fig. 5c, d).

To verify whether the NKX2–1 facilitated proliferation benefit in a serine/glycine-depleted environment is due to increased activity of the serine/glycine synthesis pathway, we tested the effect of *PSPH* shRNA-mediated knockdown on A549 vector and A549 NKX2–1 cell proliferation in normal and serine/glycine-depleted conditions (Fig. 5e). *PSPH* knockdown impaired the proliferation of vector and *NKX2–1* overexpressing cells when cultured in control medium (Fig. 5f). Interestingly, in conditions of serine/glycine depletion, *PSPH* knockdown *NKX2–1* overexpressing A549 cells entirely lost their proliferation benefit as compared to A549 vector cells.

It has previously been shown that the serine synthesis pathway is essential for the proliferation of highly aggressive brain metastatic cells [37] and breast cancer-derived lung metastases [38]. As such, we hypothesised that NKX2–1 overexpression might also confer such a metastasis benefit to cancer cells. Therefore, we first analysed whether NKX2–1 overexpression is associated with enhanced metastasising capacity in an in vitro chemotaxis assay. NKX2–1 overexpression enabled A549 cells to invade through a membrane towards foetal calf serum, whereas empty vector cells were not able to do this (Fig. 5g). Next, we analysed sequencing data of primary lung tumours and metastatic sites of NSCLC patients [39]. Confirming our in vitro observations and in line with a recently published study by Huang et al. [40], we found that *NKX2–1* amplifications are enriched in brain, lymph node and liver metastatic sites compared to the primary lung tumours (Fig. 5h). Together, these results validate that NKX2–1 overexpression provides a proliferation benefit to cells in a serine/glycine-depleted environment by activation of the serine/glycine synthesis pathway and that these cells are better adapted to invade and metastasise.

### NKX2–1 fuels redox homeostasis in vivo

**Fig. 6:**
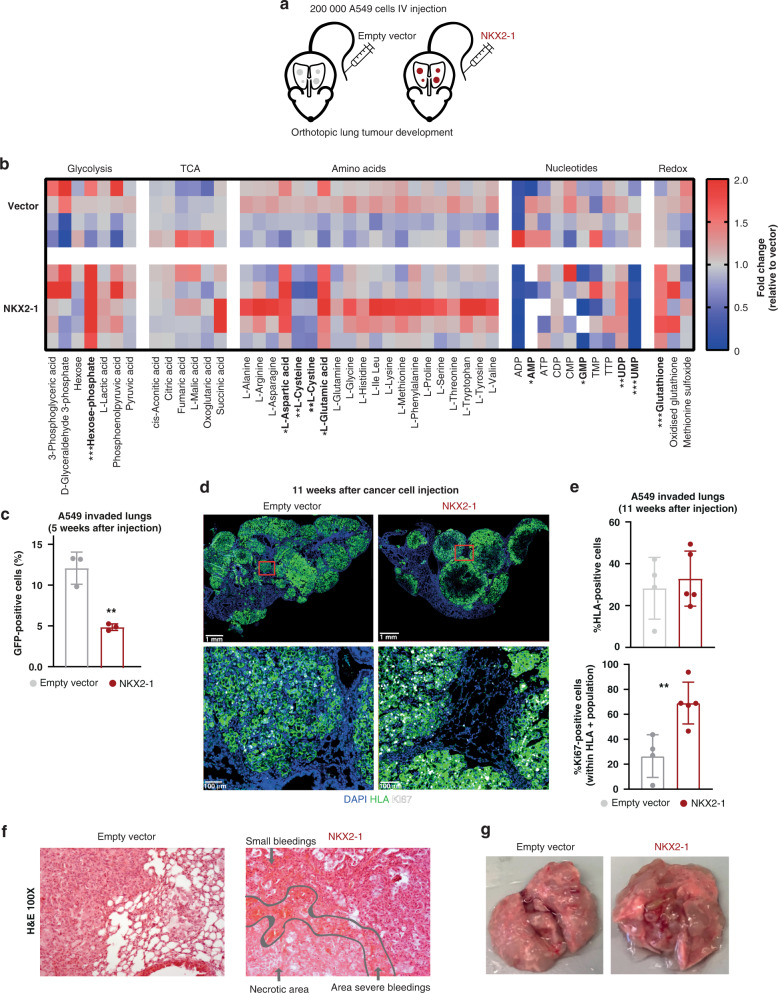
NKX2–1 expression promotes metabolic reprogramming in vivo. **a** Schematic overview of the in vivo experiment to test the effects of NKX2–1 overexpression on orthotopic lung cancer development by tail vein injection of 200,000 empty vector control or *NKX2–1* overexpressing A549 cells. **b** NKX2–1 induced fold change in blood plasma levels of tumour-bearing mice on a serine/glycine-free diet, 11 weeks after cancer cell injection (*n* = 4 for empty vector control and *n* = 5 for NKX2–1). **c** Percentage of GFP-positive cells in the lungs as determined by flow cytometry after 5 weeks of in vivo growth after intravenous injection of empty vector control or *NKX2–1* overexpressing A549 cells. The analysis was gated on viable human single cells (*n* = 3). **d** Representative images of the DAPI, Ki67 and HLA immunohistochemical stainings in lung sections of mice injected with empty vector control vs *NKX2–1* overexpressing A549 cells (*n* = 4 for empty vector control and *n* = 5 for NKX2–1). **e** Percentage of cells positive for HLA and Ki67 staining as determined by IHC quantification. **f** Representative images of H&E stainings in lung sections of mice injected with empty vector control vs *NKX2–1* overexpressing A549 cells (*n* = 4 for empty vector control and *n* = 5 for NKX2–1). **g** Representative image of lungs at the disease end stage of mice injected with empty vector control vs *NKX2–1* overexpressing A549 cells (*n* = 4 for empty vector control and *n* = 5 for NKX2–1). Data are represented as mean ± standard deviation. Individual dots represent independent observations. Statistical analysis *P value < 0.05, **P value < 0.01, ***P value < 0.001, ****P value < 0.0001. *P* values were calculated using a two-tailed Student’s *t* test.

Next, we focused on the effects of NKX2–1 expression on in vivo tumour metabolism. NSG mice were intravenously injected with *GFP*-positive vector control or *NKX2–1* overexpressing A549 cells to induce orthotopic lung cancer development (Fig. 6a). In order to allow maximum development of the NKX2–1 induced metabolic phenotype, mice received a serine/glycine-free diet after cancer cell injection. To verify whether NKX2–1 is also driving serine/glycine synthesis in vivo, we performed targeted metabolite profiling of blood plasma from these mice at 11 weeks after cancer cell injection. In line with our in vitro metabolomics experiments, no detectable differences in overall serine/glycine serum levels between control and *NKX2–1* overexpressing tumour-bearing mice were uncovered. However, as expected, since tumour cells mainly rely on the downstream metabolites generated by the serine synthesis pathway, we did detect a significant NKX2–1 associated increase in downstream GSH levels accompanied by a decrease in l-cysteine, one of the building blocks of GSH, thereby recapitulating our in vitro observations (Fig. 6b). Hence, our data support that NKX2–1 is an important regulator of redox homeostasis in vitro and in vivo.

In addition, we also monitored lung cancer progression during the experiment. In the past, NKX2–1 has often been referred to as a double-edged sword, given its described role as both a tumour suppressor as well as an oncogene [41]. We also encountered this enigma in this in vivo experiment. When we compared the amount of GFP-positive A549 cells present in the lungs at an early timepoint (5 weeks) after cancer cell injection, we observed a twofold lower engraftment of *NKX2–1* overexpressing cells in the lungs compared to empty vector control cells, indicating rather a tumour suppressive role of NKX2–1 (Fig. 6c). Remarkably, 11 weeks after cancer cell injection, mice injected with *NKX2–1* overexpressing A549 cells showed similar HLA staining of the lungs indicating a phenotypic switch of NKX2–1 cells in the tumour. This finding was supported by elevated expression of the Ki67 proliferation marker, pointing towards a rather oncogenic role of NKX2–1 (Fig. 6d, e). Moreover, pathologic examination of H&E stained tumours, revealed severe bleedings and necrosis in the lungs infiltrated by NKX2–1 tumours, but not in vector control tumours, highlighting a more aggressive phenotype of NKX2–1 tumours. (Fig. 6f, g). Yet, NKX2–1 tumours do not show altered endothelial CD31/PECAM-1 staining patterns (Supplementary Fig. 9). Overall, these in vivo data together with the patient observations (Fig. 5i) strongly indicate that NKX2–1 overexpression mainly contributes to cancer progression and spreading when the tumour size has become substantial and availability for nutrients such as serine and glycine is limited.

### NKX2–1 overexpressing cells are sensitised to SHMT inhibitor sertraline and the clinically used chemotherapeutic etoposide

Despite the improved outcomes of T-ALL and LUAD patients over the last decade, treatment-related toxicity and therapy resistance remain major problems [23, 25]. Therefore, targeted treatments that specifically interfere with the cellular processes that are deregulated in cancer cells, such as an increase in serine/glycine metabolism, are desirable. Our lab recently identified the widely clinically used anti-depressant sertraline as a potent SHMT inhibitor that selectively inhibits proliferation of serine synthesis addicted breast cancer and RPL10 R98S T-ALL cells [5].

**Fig. 7:**
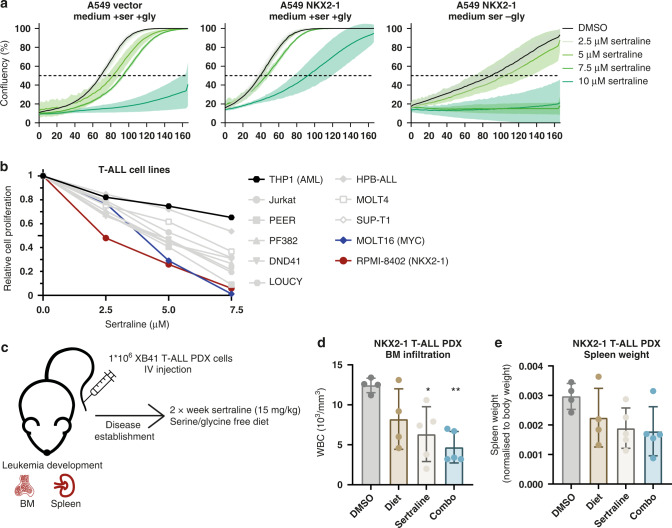
NKX2–1 expressing cells are sensitive to sertraline treatment. **a** Cell confluency over time of A549 cells in the presence of increasing sertraline concentrations in DMEM with or without serine/glycine. A representative experiment (*n* = 3 biological replicates) with three technical replicates is shown. **b** Relative cell proliferation of T-ALL cells after 72 h of culturing with increasing concentrations of sertraline in control RPMI-1640 (*n* ≥ 3). **c** Schematic overview of the in vivo experiment to test the effects of sertraline treatment (15 mg/kg, twice a week) and/or a serine/glycine-free diet on T-ALL disease progression following intravenous injection of 1*10^6^ NKX2–1-positive XB41 PDX T-ALL cells. **d** White blood cell counts (WBC) measured at disease end stage in a bone marrow (BM) sample taken from the femur of the leukaemic mice (*n* = 4 for DMSO and diet group; *n* = 5 for sertraline and combo group). **e** Spleen weight normalised to body weight of the leukaemic mice measured at disease end stage (*n* = 4 for DMSO and diet group; *n* = 5 for sertraline and combo group). Data are represented as mean ± standard deviation. Individual dots represent independent observations. Statistical analysis *P value < 0.05, **P value < 0.01, ***P value < 0.001, ****P value < 0.0001. *P* values were calculated using a two-tailed Student’s *t* test.

We first examined the effect of sertraline on the cell proliferation of A549 cells and found that *NKX2–1* overexpressing A549 cells were sensitised to sertraline in serine/glycine-depleted conditions (Fig. 7a). Next, we examined the effect of sertraline on the proliferation of a panel of 10 T-ALL cell lines and 1 AML cell line, the latter as a negative control. As anticipated due to generalised serine/glycine synthesis pathway upregulation in T-ALL, the proliferation of most T-ALL cell lines was inhibited by sertraline (Fig. 7b and Supplementary Fig. 10a). Strikingly, among all T-ALL cell lines tested, the only NKX2–1 expressing cell line RPMI-8402 was most sensitive to sertraline treatment. Moreover, the T-ALL cell line second most sensitive to sertraline, MOLT-16, carries a MYC amplification, which has been identified as a serine synthesis inducing genetic alteration [8].

To validate these encouraging in vitro results in vivo, we intravenously injected an NKX2–1 positive T-ALL PDX (XB41) into NSG mice. Since the effect of serine synthesis inhibitors can significantly be enhanced in combination with a serine/glycine-free diet [42], we tested the effects of sertraline in combination with a serine/glycine-free diet in this in vivo model. After bone marrow engraftment and disease progression, mice received sertraline (15 mg/kg, twice a week) and/or serine/glycine-free diet (Fig. 7c). At disease end stage, sertraline had effectively decreased disease progression shown by decreased spleen size and less infiltration of leukaemia cells in the bone marrow of the sertraline treatment group compared to control (Fig. 7d, e). Strikingly, the combination of a serine/glycine-free diet and sertraline even more drastically suppressed disease progression (Fig. 7d, e).

**Fig. 8:**
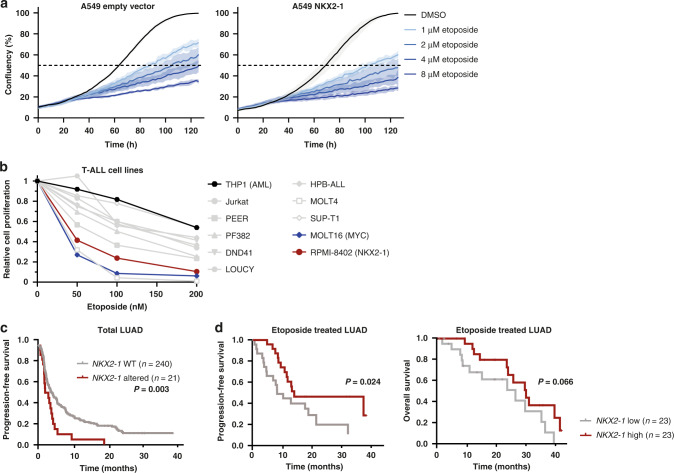
NKX2–1 expression sensitises cells and LUAD patients to etoposide treatment. **a** Cell confluency over time of A549 cells in the presence of increasing etoposide concentrations. A representative experiment (*n* = 3 biological replicates) with three technical replicates is shown. **b** Relative cell proliferation of T-ALL cells after 72 h of culturing with increasing concentrations of etoposide in control RPMI-1640 (*n* ≥ 3). **c** Kaplan–Meier analysis of the progression-free survival of LUAD cancer patients with and without *NKX2–1* genetic alteration (mutations and amplifications, *n* = 21) versus *NKX2–1* WT patients (*n* = 240) (cBioportal NSCLC datasets combined, selecting for LUAD patients). *P* values were calculated using a Log-rank (Mantel–Cox) test. **d** Kaplan–Meier analysis of overall survival and progression-free survival equally divided in high versus lower 50% *NKX2–1* expression levels in Stage III LUAD patients (*n* = 46). Data were obtained from the cBioportal platform (TCGA dataset). *P* values were calculated using a Log-rank (Mantel–Cox) test. Data are represented as mean ± standard deviation. Individual dots represent independent observations. Statistical analysis *P value < 0.05, **P value < 0.01, ***P value < 0.001, ****P value < 0.0001.

Next, since nucleotide synthesis was strongly induced downstream of NKX2–1-driven serine/glycine synthesis (Fig. 4e–g), we reasoned that it may also be beneficial to target this process. Etoposide, clinically used for the treatment of T-ALL and LUAD, is a topoisomerase II inhibitor and as such restrains DNA replication, which requires nucleotide synthesis. Interestingly, the proliferation of *NKX2–1* overexpressing A549 cells was significantly more inhibited by etoposide compared to control cells, both in control and serine/glycine-depleted conditions (Fig. 8a). While most of the tested T-ALL cell lines were sensitive to etoposide, the NKX2–1 expressing RPMI-8402 cell line again emerged as one of the most sensitive cell lines (Fig. 8b and Supplementary Fig. 10b).

Lastly, we screened for the impact of *NKX2–1* genetic alterations on LUAD progression and response to therapy. We observed that LUAD patients with a genetic *NKX2–1* alteration present a reduced progression-free survival (Fig. 8c). However, LUAD patients all receive different treatment regimens according to their tumour stage. Retrospective analysis of sertraline was not possible due to lack of sertraline-treated patients. Using publicly available datasets, we selected the LUAD patients that specifically received etoposide treatment, as is the case in Stage III patients [43]. In this subset of LUAD patients (*n* = 46), we analysed the link between NKX2–1 expression and patient’s survival rates. Remarkably, we show a better progression-free-survival for patients with high *NKX2–1* mRNA (50% highest) expression levels compared to patients with low *NKX2–1* expression levels (50% lowest), and a trend for a higher overall survival probability (Fig. 8d). In summary, these results illustrate the dependence of NKX2–1 expressing cells on the serine/glycine synthesis pathway for nucleotide metabolism and highlight these processes as promising therapeutic targets in NKX2–1-driven cancers.

## Discussion

De novo serine/glycine synthesis is hyperactivated in numerous cancer subtypes, including T-ALL and LUAD [12, 44]. Although several mechanisms driving this hyperactivation have been identified, it remains unexplained in a large fraction of tumours [45]. To address this, we analysed public RNA- and ChIP-Seq data of T-ALL and LUAD patients, which identified NKX2–1 as a potential novel direct regulator of serine/glycine synthesis enzymes. Further characterisation by metabolic profiling and in vivo experiments identify NKX2–1 as a novel oncogenic driver of serine/glycine synthesis addiction in cancer.

Besides direct regulation of de novo serine/glycine synthesis, the cyclin D3:CDK4/6 complex has been described as an important indirect regulator in T-ALL through inhibition of glycolysis enzymes 6-phosphofructokinase (PFKP) and pyruvate kinase M2 (PKM2). Blocking these enzymes enables the leukaemic cells to redirect glycolytic intermediates into the pentose phosphate (PPP) and serine pathway to supply the cells with GSH and NADPH [46]. Yet, this mechanism may be more difficult to target, as cells can also redirect to the PPP pathway, creating less dependency on serine and glycine metabolism. This may explain why most T-ALL cell lines, which typically show Cyclin D3:CDK4/6 complex activation, are less sensitive to sertraline and etoposide as compared to the NKX2–1-positive RPMI-8402 cell line. Furthermore, NOTCH1 is mutated in the majority of T-ALL patients. As MYC is controlled by NOTCH1 and MYC can regulate expression of the serine/glycine synthesis enzymes [8, 47], we analysed the impact of NOTCH1 mutations on mRNA expression levels of serine/glycine synthesis enzymes in T-ALL patients [15]. NOTCH1-mutated tumours did show higher expression of serine/glycine metabolism enzymes PHGDH, SHMT1 and SHMT2 (Supplementary Fig. 11a). However, when we group samples according to both NOTCH1 and NKX2–1 mutational state, it is clear that NKX2–1-positive NOTCH1-mutated patients have significantly elevated expression levels of the serine/glycine synthesis enzymes compared to NKX2–1-negative NOTCH1-mutated patients, supporting that NKX2–1 is thus an additional and novel inducer of serine/glycine synthesis enzyme expression (Supplementary Fig. 11b).

Although our RNA-Seq analysis identified NEPC as a cancer subtype in which *NKX2–1* mutations are associated with increased *PSPH* mRNA expression (Fig. 1f), we did not further investigate this cancer subtype, due to the limited availability of prostate cancer models with neuroendocrine origin. Nonetheless, serine/glycine synthesis pathway upregulation has been described before in NEPC as an indirect result of protein kinase C λ/ι downregulation [48].

We showed that NKX2–1 binding to the regulatory regions of serine/glycine synthesis enzyme genes increased in response to serine/glycine starvation. In line with this, ATF3 only promotes serine/glycine synthesis under serine-restricted conditions [49]. Furthermore, MYC-mediated activation of the serine synthesis pathway almost exclusively occurs in response to glucose or glutamine starvation and myeloid leukaemic cells become more dependent on the serine synthesis pathway in fructose-rich conditions compared to glucose-rich conditions [8, 50]. These findings highlight the importance of the microenvironmental condition to induce metabolic rewiring in times of need. In fact, standard cell culture media do not recapitulate the complexity of a human tumour microenvironment and adaptations to better mimic the physiological situation are needed to better understand metabolic rewiring in cancer. This prompts us to consider that besides environmental serine/glycine concentration, other variables probably also are important to induce the NKX2–1 dependent transcriptional programme, indicating that our observations might be an underestimation of the human situation, especially when leukaemic stem cells encounter hypoxic niches in the bone marrow and subsequent limited availability of many nutrients.

To our surprise, *NKX2–1* overexpression in A549 cells led to increased intracellular triglycerides levels (Fig. 4j). Correspondingly, we observed that patients with serine synthesis addicted RPL10 R98S mutated T-ALL present a trend for increased serum triglyceride levels compared to patients with RPL10 WT T-ALL (*n* = 4 R98S and *n* = 3 WT cases *P* = 0.103 (Supplementary Fig. 12)). Previously, an association between increased methylation levels of the *PHGDH* promoter in circulating DNA and a decrease in blood triglyceride levels was observed, but a more in-depth connection between serine and triglycerides has not been established [51]. Classically, high triglyceride levels have been reported to be associated with cancer initiation [52]. However, based on our findings, we speculate that triglyceride might have a role in supporting cancer progression as well.

NKX2–1 belongs to the NKL homeobox gene family and it has been suggested that these genes share similar downstream effects that can promote tumorigenesis [30]. Interestingly, NKX3-1 has been identified to protect prostate cancer cells from oxidative stress [53]. Therefore, it will be worth to explore whether our finding that NKX2–1 positive cancers are sensitive to sertraline and etoposide can be extended to cancer types that overexpress other NKL homeobox genes such as NKX2-2 in Ewing’s sarcoma [54], NKX2-5 in ovarian yolk sac tumours [55] and NKX3-1 in prostate cancer and ductal/lobular breast carcinomas [56].

Interestingly, redox stress is an important factor that limits the survival of metastatic cancer cells and treatment of mice with antioxidants increases the metastatic disease burden [57, 58]. Within this context, we found that GSH serum levels are increased in NKX2–1 tumour-bearing mice compared to control tumour-bearing mice (Fig. 6b). In addition, we discovered that NKX2–1 overexpression enables cancer cells to invade and that NKX2–1 genetic alterations are enriched in metastatic sites compared to primary tumours (Fig. 5g, h). Therefore, we propose that NKX2–1 overexpression could represent a survival mechanism of cancer cells to manage oxidative stress during metastasis. In contrast, in early-stage cancers, oxidative stress is known to promote cancer progression by causing DNA damage, which contributes to the formation of oncogenic mutations [57]. As such, at early-disease stage, NKX2–1 overexpression might limit ROS levels needed to acquire additional oncogenic driver mutations and in this way function as a tumour suppressor, which could explain our observation that NKX2–1 overexpressing A549 cells engrafted to a lesser extent in the lungs at early-disease stage (Fig. 6c). Moreover, these findings could add to a better understanding of the cellular contexts in which NKX2–1 functions as either an oncogene or tumour suppressor.

The identification of specific mutations and their association with the deregulation of cellular processes in distinct cancer types is an important step in the road towards personalised medicine. Patient stratification is key to predict the most effective treatment options. We identified that NKX2–1 overexpressing cells are more sensitive to both sertraline and etoposide. Interestingly, we observed that A549 cells were mainly sensitive to sertraline in conditions of low serine/glycine availability (Fig. 7a) and that the in vivo anti-tumorigenic of sertraline on leukaemia progression could be enhanced by a serine/glycine-free diet (Fig. 7c–e) suggesting that the application of sertraline for cancer treatment might mainly be beneficial either in conditions of low serine/glycine availability or in conditions of increased serine/glycine synthesis demand. The latter can be promoted by, for example, combining sertraline with drugs that inhibit mitochondrial metabolism [5] or mTORC1 [59].

In conclusion, we identified the homeodomain transcription factor NKX2–1 as a novel direct oncogenic inducer of serine/glycine synthesis enzymes in cancer, and we characterised downstream consequences on cellular nucleotide, redox and lipid metabolism and DNA methylation. Our findings identify NKX2–1 tumours as an extra subset of cancers that can benefit from the therapeutic targeting of serine/glycine synthesis hyperactivation.

## Materials and methods

### Analysis of public data

Analysed RNA-Seq data for T-ALL (*n* = 264) were from [15], for NEPC (*n* = 49) from [21] and for LUAD (*n* = 566) from TCGA (cancergenome.nih.gov, Lung Adenocarcinoma PanCancer Atlas dataset). Only samples without known genetic alterations (amplifications, deletions, point mutations and translocations) in *PHGDH*, *PSAT1*, *PSPH*, *SHMT1* and *SHMT2* were included for further analysis.

NKX2–1 ChIP-Sequencing data in A549 cells were from [27] and were visualised with the Cistrome web browser [60]. H3K27ac and ATAC-seq peaks were obtained from the ENCODE project [28] and visualised in the UCSC genome browser [61].

Kaplan–Meier curves were generated with SPSS. Progression-free survival considering all treatment regimens was analysed using combined datasets of NSCLC patient samples from [62–65], and selecting for LUAD cases. NKX2–1 WT (*n* = 240) vs NKX2–1 altered (*n* = 21; mutation, amplification) were compared (Fig. 8e). Etoposide-specific progression-free and overall survival data were from (cancergenome.nih.gov, Lung Adenocarcinoma PanCancer Atlas dataset, *n* = 566 for total dataset; *n* = 46 for etoposide treated patients). Median expression was used as cut-off for high versus low NKX2–1 expression groups (Fig. 8f). Datasets were downloaded from cBioportal. Statistical analysis was performed with Log-rank (Mantel–Cox) test.

NSCLC sequencing data according to tissue sampling site were from [39] and downloaded from cBioportal. Statistical analysis was performed with Fisher’s exact test.

### Cell culture

A549 (ACC 107), Ba/F3 (ACC 300), DND41 (ACC 525), RPMI-8402 (ACC 290) and 293T (ACC 635) cell lines were obtained from the German Collection of Microorganisms and Cell Cultures GmbH (DSMZ). NCI-H125 were historically obtained from Molecular Biology Dept. at Maastricht University (P14). A549 and 293T cells were cultured in DMEM (Gibco) with 10% foetal calf serum (FCS) (Gibco). For assays in serine/glycine-depleted condition, cells were plated in DMEM without serine and glycine (US Biological life Sciences, D9802-01), supplemented with 4.5 g/L glucose (Sigma-Aldrich), 3.7 g/L sodium bicarbonate (Sigma-Aldrich), GlutaMAX™ (Gibco) and 10% dialysed serum (Life Technologies, A3382001). RPMI-8402 and NCI-H125 were cultured in RPMI-1640 (Gibco) with 10% FCS. Ba/F3 cells were cultured in RPMI-1640 (Gibco) supplemented with 10% FCS (Gibco) and IL-3 (Miltenyi, 10 ng/mL). DND41 cells were cultured in RPMI-1640 (Gibco) supplemented with 20% FCS (Gibco). For experiments in serine/glycine-depleted condition, cells were cultured in serine–glycine-glucose-free RPMI-1640 (Teknova) supplemented with 0.5 mg/ml or 2 mg/mL glucose (Sigma-Aldrich) and with 10% or 20% dialyzed serum (Life Technologies, A3382001). In experiments where conditions with and without serine and glycine were compared, serine and glycine (Sigma-Aldrich) were supplemented to the serine/glycine-free culture media as indicated in the dedicated figures (400 µM). Cultured cells were all mycoplasma negative.

### Viral vectors

A codon-optimised sequence of human NKX2–1 encoding cDNA sequence was ordered as gBLOCK and inserted into a pMSCV-IRES-GFP vector (Supplementary Table 1). shRNA sequences targeting NKX2–1 (Supplementary Table 1) were cloned into pLKO.1 mCherry (Addgene #128073). shPSPH-pLKO.1 was described before [12]. Retroviral particles were generated by co-transfection of the pMSCV-IRES-GFP plasmid with packaging plasmid pCL-Ampho (Bio-Techne), for human cell transduction, or ecopac (pIK6.1MCV.ecopac.Utd) for murine cell transduction, into 293T cells using GeneJuice Transfection Reagent (Merck Millipore). Lentiviral particles were generated by transfecting the pLKO.1 mCherry plasmid into 293 T cells using a VSV-G envelope and psPAX2 packaging plasmid. Cells were incubated overnight with viral supernatants in the presence of 1 µg/mL polybrene (Merck Millipore), after which stably transduced cells were expanded. Fluorophore-expressing cells were sorted for GFP and/or mCherry on a S3 cell sorter (Bio-Rad) to obtain >80% vector-enriched cultures. While culturing the cells, the fraction of transduced cells was regularly verified on a MACS VYB flow cytometer (Miltenyi).

### CRISPR-Cas9 genome editing

RPMI-8402 NKX2–1 KO cells were generated by CRISPR-Cas9 genome editing. RPMI-8402 cells were electroporated (6 square wave pulses, 0.1 ms interval, 150 V) with an SpCas9-2A-GFP-encoding pX458 vector (Addgene #48138) containing an NKX2–1 targeting gRNA (5’-AAGATGTCAGACACTGAGAA-3’). Following electroporation, cells were incubated for 48 h in recovery medium (RPMI-1640 supplemented with 100× non-essential amino acids (Gibco) and 1 mM sodium pyruvate (Gibco)), followed by sorting for GFP (BD FACS Aria III). Cells were both single-cell sorted in 96-well plates and bulk-sorted. Bulk-sorted cells were subsequently grown to single-cell colonies in ClonaCell™-TCS (Stem Cell Technologies, # 03814). Growing clones were expanded and screened for the desired modification using the Synthego ICE analysis tool (v3.0) on Sanger sequencing results of the PCR-amplified NKX2–1 locus (Fw: 5’-CCGTTACGTGTACATCCAAC-3’; Rv: 5’-CTGTTCCTCATGGTGTCCTG-3’).

### ChIP-qPCR

SimpleChIP Plus Sonication Chromatin IP Kit (Cell Signaling) was used to perform all ChIP experiments using either anti-TTF1 (Abcam, ab76013) or anti-IgG (Cell Signaling, #2729) antibodies according to manufacturer’s instructions using Dynabeads Protein G (Invitrogen) magnetic beads. Cells were cross-linked for 10 min by adding formaldehyde (final concentration 1%) to a cell pellet (suspension cells) or culture flask (confluent cells) after washing the cells in PBS. Crosslinking was quenched by adding 1.25 M glycine. Then, cells were washed twice in ice-cold PBS. Sonication of the samples was performed on a Bioruptor NGS (Diagenode) for ten cycles of 30 s activity −30 s rest. DNA was purified using PCR purification columns (Qiagen) and served as template in qPCR (primer sequences, Supplementary Table 2).

### Motif enrichment

Cluster-Buster (cBust) [29] is a tool to better define the occurrence of clusters of pre-specified motifs in DNA sequences, as individual DNA motifs are often too short and degenerate. The cBust tool was used to identify clustered motif occurrences of previously described NKX motifs in the promoter of *PHGDH*, *PSAT1*, *PSPH* and *SHMT2* and putative enhancer regions of *PSPH*. The log-likelihood score thresholds were set at 3 for clusters (-c 3). This resulted in a.gff file that was visualised using TOUCAN (https://toucanjs.aertslab.org/toucanjs.html) [66] without showing the individual motifs. Since all 14 NKX genes (*NKX1-1*, *NKX1-2*, *NKX2–1*, *NKX2-2*, *NKX2-3*, *NKX2-4*, *NKX2-5*, *NKX2-6*, *NKX2-8*, *NKX3-1*, *NKX3-2*, *NKX6-1*, *NKX6-2*, *NKX6-3*) belong to the same family of NKL homeobox genes, and are thus structurally similar and have comparable binding motifs [67], we included multiple NKX motifs such as motifs for NKX2–1, NKX2-2, NKX2-3, NKX2-5 and NKX2-8 in our analysis (Supplementary Fig. 2d). Position weight matrices of the different motifs were visualised by the R-package seqLogo (Version 1.60.0). The motifs included per NKX2 CRM cluster are defined in Supplementary Table 4. Clusters were defined per genomic region.

### Quantitative RT-qPCR

RNA was extracted using the RNeasy kit (Qiagen) followed by cDNA synthesis of 500 ng RNA using random hexamer primers, and a mix of ribonuclease inhibitors, dNTPs and GoScript reverse transcriptase (Promega). Serine/glycine synthesis enzyme mRNA expression levels together with ACTB1 as a reference gene, were analysed in quadruplicate using SYBR Green-based qRT-PCR on a CFX Connect instrument (Bio-Rad Laboratories). Relative mRNA expression from at least three technical replicates per biological repeat was determined using the ΔΔCt method (primer sequences, Supplementary Table 2). At least three biological repeats were included in the figures. For NCI-H125 experiments, only samples with at least 20% *NKX2–1* knockdown on RNA level were considered.

### Immunoblotting

Cells were lysed (cell lysis buffer 6, R&D Systems) and denatured in 1× Laemmli sample buffer (Bio-Rad) containing 2-mercaptoethanol (Sigma-Aldrich). Proteins were separated on Criterion Tris-Glycine eXtended gels (Bio-rad), transferred to PVDF membranes, and incubated with primary and secondary antibodies (Supplementary Table 3). Proteins were visualised using chemiluminescence on an Azure C600 (Azure Biosystems). Quantification was performed using LI-COR Image Studio Lite software version 5.2. Vinculin and β-actin were used to normalise for protein input. Immunoblots were performed on samples from at least three biological repeat experiments.

### ^13^C tracer analysis

Ba/F3 labelling was performed in serine–glycine-glucose-free RPMI-1640 (Teknova) supplemented with ^13^C_6_-glucose (Sigma-Aldrich) to a final concentration of 1 mg/mL and with 10% dialyzed serum (Life Technologies, A3382001) for 42 h. RPMI-8402 and NCI-H125 labelling was performed 48 h after *NKX2–1* knockdown in serine–glycine–glucose-free RPMI-1640 (Teknova) supplemented with ^13^C_6_-glucose (Sigma-Aldrich) to a final concentration of 2 mg/mL and with 10% dialyzed serum (Life Technologies, A3382001) for 48 h. A549 labelling was performed in DMEM (US Biological life Sciences, D9802-01), supplemented with 4.5 g/L ^13^C_6_-glucose (Sigma-Aldrich), 3.7 g/L sodium bicarbonate (Sigma-Aldrich), GlutaMAX™ (Gibco) and 10% dialyzed serum for 48 h. Samples were processed as follows, 10 µl of the cell culture medium was added to 990 µl ice-cold myristic acid containing 80% methanol-based extraction buffer, and cells were washed in 0.9% NaCl. Cells were lysed in 800 µl or 300 µl of extraction buffer and extracted suspensions were stored at −80 °C for one day, centrifuged and sent for mass spectrometry analysis (VIB/KU Leuven metabolomics core facility). Mass spectrometry was performed as described previously [12].

### Lipidomics

A549 cells were cultured in DMEM (US Biological life Sciences, D9802-01), supplemented with 4.5 g/L ^13^C_6_-glucose (Sigma-Aldrich), 3.7 g/L sodium bicarbonate (Sigma-Aldrich), GlutaMAX™ (Gibco) and 10% dialyzed serum for 4 h prior to pellet collection. Lipidomics analysis was performed by the KU Leuven Lipometrix facility, as described previously [68].

### Methylation sequencing

NCI-H125 pellet collection was performed 48 h after *NKX2–1* knockdown after culturing the cells for 4 h in serine–glycine-glucose-free RPMI-1640 (Teknova) supplemented with 2 mg/ml glucose (Sigma-Aldrich) and with 10% dialyzed serum (Life Technologies, A3382001). DNA was extracted using the Nucleopsin Tissue kit (Macherey-Nagel, 10498961) and sheared with a Bioruptor NGS (Diagenode) for seven cycles of 30 s activity—90 s rest. Next, enzymatic conversion of cytosine in the sheared DNA was performed with the NEBNext Enzymatic Methyl-seq conversion module (New England Biolabs, E7125S) according to the manufacturer’s instructions. Afterwards, sequencing (paired end, 150 bp) was performed on an Illumina Novaseq6000 instrument with chemistry V1.5. Sequencing reads were trimmed using TrimGalore (version 0.4.1), and the trimmed FASTQ files were subsequently mapped on a bisulfite-converted human genome (GRCh37) using Bismark (version 0.19.0) [69]. Coverage files were extracted using Bismark’s methylation extractor to quantify the degree of methylation of each CpG position. Next, differential methylation analysis was performed using Methylkit [70] with overdispersion correction and by tiling windows analysis using a step size and length of 250 bp. Samples from the same group (scramble, shNKX2-1 #1 or shNKX2-1 #2) were pooled together. Significantly differentially methylated regions (DMRs) were selected using a percent methylation difference larger than 20% and a *q*-value smaller than 0.01. We annotated the DRMs based on their overlap with promoter regions, defined as regions starting 1500 base pairs before the transcription start site and ending 500 base pairs after. Only genes with more than 100 CpG counts in the overlapping DRM and more than 30% methylation difference were reported. As we obtained the best *NKX2–1* knockdown with shNKX2-1 hairpin #1, these results are the most reliable. All statistical analyses were performed in R, version 4.2.0.

### Incucyte

For cell proliferation assays, A549 cells were plated at 2000 (A549 NKX2–1 cells) or 2500 (A549 empty vector cells) cells per well and NCI-H125 at 5000 cells per well in 96-well TPP plates in six replicates to obtain a starting confluence of 10%. Photomicrographs were taken every 2 h using an Incucyte live cell imager (Sartorius), and the confluence of the cultures was determined using Incucyte software. For invasion assays, A549 cells were plated at 750 cells per well in DMEM containing 0.5% FCS in the upper reservoir of a ClearView migration plate. The bottom reservoir was filled with 200 µl DMEM containing 10% FCS. Whole-well images of cells on both sides of the membrane either from the top or from the bottom of the ClearView migration plate were captured every 2 h. Cell invasion was reported as an increase in the area on the bottom side of the membrane for adherent A549 cells that migrated down the pores to the reservoir wells.

### Flow cytometry

Total cell counts were measured by FSC/SSC analysis on a Guava easyCyte HT (Merck) and viability was determined by Annexin V-PE (IQ products, 1:100) and Zombie Aqua (Biolegend, 1:1000) staining. Viable cell counts were defined as the proportion of total cell counts staining negative for Annexin V and Zombie aqua. ROS levels were determined using CellROX Deep Red Reagent or CellROX Green Reagent (Thermo Fisher Scientific) according to the manufacturer’s instructions. All samples were measured using a MACSQuant VYB (Miltenyi) or a FACS Canto II (Beckton Dickinson) flow cytometer, and data were analysed using FlowJo software.

### Xenografts in NOD-SCID/IL2γ^−/−^ (NSG) mice

Animal experiments were approved by the KU Leuven animal ethics committee (ECD approval P035/2020). NSG breeding pairs were recently purchased from Charles River laboratories and were bred at the animal facility at Gasthuisberg (Leuven) to establish a colony. For the orthotopic model, A549 cells (200 000) were injected in the tail veins of 6–8-week-old NSG mice of both sexes to form lung tumours. Animals were randomly distributed into different groups. After cancer cell injection, mice received an experimental diet: Mod testdiet 5cc7 w/no added serine or glycine (T-5BJX-1817070-203, TestDiet). Animals were sacrificed after 5 or 11 weeks by terminal cardiac bleeding under pentobarbital (150 mg/kg) anaesthesia to obtain blood plasma. Plasma samples were analysed by targeted mass spectrometry. Dissected lungs from sacrificed animals were preserved in formaldehyde, followed by paraffin embedding for H&E staining and immunohistochemistry.

For in vivo treatment studies, 6–8-week-old mice of both sexes were injected with 10^6^ XB41 T-ALL patient cells via intravenous tail vein injection. Animals were randomly distributed into different groups. When engraftment was established 1 week after cell injection, mice were treated for 4 consecutive weeks. Sertraline (15 mg/kg) was delivered through intraperitoneal injection twice weekly. The experimental diet: Mod testdiet 5cc7 w/no added serine or glycine (T-5BJX-1817070-203, TestDiet) was given continuously starting 1 week after cancer cell injection. The appropriate sample size was estimated upon earlier performed experiments [12].

### T-ALL patient samples and patient-derived xenografts (PDX)

Experiments on human samples were approved and supervised by the UZ Leuven ethical committee. Informed consent was obtained from all subjects. Peripheral blood samples obtained at diagnosis were collected from children diagnosed with T-ALL at University Hospital Leuven, as approved by the local Ethical Committee (S54608).

### Immunofluorescence

The following antibodies were used for detecting the respective proteins: anti-HLA (rabbit, 1:1500, Abcam, ab52922) and anti-Ki67 (rabbit, 1:1000, Fisher Scientific, RM-9106-S0). The Akoya Opal Polaris 7 Colour Automation IHC Detection Kit (Akoya, NEL871001KT) was used for tyramide signal amplification according to the manufacturer’s protocol. For the introduction of the secondary-HRP the Envision + /HRP goat anti-Rabbit (Dako Envision + Single Reagents, HRP, Rabbit, Code K4003) was used for all antibodies raised in rabbit. The various proteins were detected by first using the OPAL 520 (HLA), and at last OPAL 780 (Ki67) reagents according to the manufacturer’s protocol. CD31/PECAM-1 (Cell signaling #77699) staining’s were performed according to the manufacturers’ protocol. Pathologic examination of tumours by H&E was performed by an experienced mouse pathologist, Marion Gijbels at Maastricht University.

### Microscope image acquisition and image processing

Images were acquired on the Akoya Vectra Polaris using a ×20 objective without binning. Image analysis was processed in QuPath v.0.2.3 [71]. Cell detection was conducted using QuPath’s built-in “Positive cell detection”. For each sample, the total number of cells and cells positive for Ki67 or HLA was assessed.

### Compounds

Sertraline (Sigma-Aldrich, #S6319) and Etoposide (Selleckchem, S1225) were dissolved in DMSO and stored at −20 °C and −80 °C. Hydrogen peroxide was purchased from Sigma-Aldrich (H1009).

### Statistics

All statistical analyses were performed using Prism 7/8 (GraphPad). A two-tailed Student’s *t* test or nested *t* test was used to compare empty vector and NKX2–1 conditions for all in vitro and in vivo measurements based upon *F*-test for Equality of Variances unless otherwise stated. All experiments were performed at least three times.

### Figures

For Figs. 6 and 7 and Supplementary Fig. 9, we made use of graphics designed by Alina Oleynik, Julie Ko and Hui wen from NounProject.com.

## Supplementary information

Supplementary figures and data tables

## Data Availability

The data generated in this study are available within the article and its supplementary data files.
