# Supplementary material for: Transcription factor NKX2–1 drives serine and glycine synthesis addiction in cancer
Source: Br J Cancer. 2023 Mar 17;128(10):1862–78. doi: 10.1038/s41416-023-02216-y (PMC10147615; doi:10.1038/s41416-023-02216-y)
Supplement: Supplementary file 1 — Supplementary figures and data tables [file 41416_2023_2216_MOESM1_ESM.docx]

**Supplementary data file**

**TRANSCRIPTION FACTOR NKX2-1 DRIVES SERINE AND GLYCINE SYNTHESIS ADDICTION IN CANCER**

**Elien Heylen** ^1,2^, Paulien Verstraete ^1,2^, Linde Van Aerschot ^1,2^, Shauni Geeraerts ^1,2^, Tom Venken ^3,4^, Kalina Timcheva ^1,2^, David Nittner ^5,6^, Jelle Verbeeck ^1,2^, Jonathan Royaert ^1,2^, Marion Gijbels ^7,8^, Anne Uyttebroeck ^2,9^, Heidi Segers ^2,9^, Diether Lambrechts ^3,4^, Jan Cools ^2,4,10^, Kim De Keersmaecker ^1,2*^, Kim R Kampen ^1,2,11*^

^1^ Laboratory for Disease Mechanisms in Cancer, Department of Oncology, KU Leuven

^2^ Leuven Cancer Institute (LKI), Leuven, Belgium.

^3^ Laboratory for Translational Genetics, Department of Human Genetics, KU Leuven, Leuven, Belgium

^4^ Center for Cancer Biology, VIB, Leuven, Belgium

^5^ Histopathology Expertise Center, VIB-KU Leuven Center for Cancer Biology, VIB, Leuven, Belgium

^6^ Department of Oncology, KU Leuven, Leuven, Belgium

^7^ Department of Pathology, GROW School for Oncology and Reproduction, Maastricht, The Netherlands.

^8^ Department of Medical Biochemistry, Experimental Vascular Biology, Amsterdam Cardiovascular Sciences, Amsterdam Infection and Immunity, Amsterdam UMC, Amsterdam, Netherlands

^9^ Paediatric Haematology and Oncology, University Hospitals Leuven, Department of Oncology, KU Leuven

^10^ Center for Human Genetics, KU Leuven, Leuven, Belgium

^11^ Maastricht University Medical Centre, Department of Radiation Oncology (MAASTRO), GROW School for Oncology and Reproduction, Maastricht, The Netherlands.

* These authors jointly supervised this work: Kim De Keersmaecker and Kim R. Kampen. ✉e-mail: [kim.dekeersmaecker@kuleuven.be](mailto:kim.dekeersmaecker@kuleuven.be) ; [k.kampen@maastrichtuniversity.nl](mailto:k.kampen@maastrichtuniversity.nl)


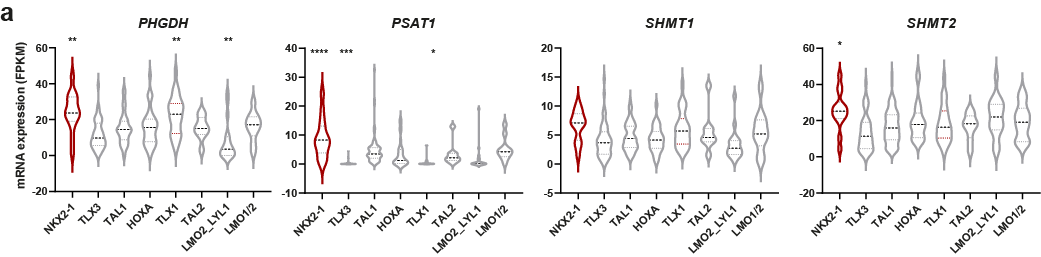


**Supplementary figure 1.** **NKX2-1 overexpression is associated with elevated mRNA expression of serine and glycine synthesis enzymes in T-ALL.**

**a)** *PHGDH, PSAT1, SHMT1* and *SHMT2* mRNA expression levels according to genetic subgroup in 264 T-ALL patients ^1^ (Dunnett's multiple comparisons test).

All violin plots show the median and quartiles. Statistical analysis *p-value < 0.05, **p-value < 0.01, ***p-value < 0.001.

**
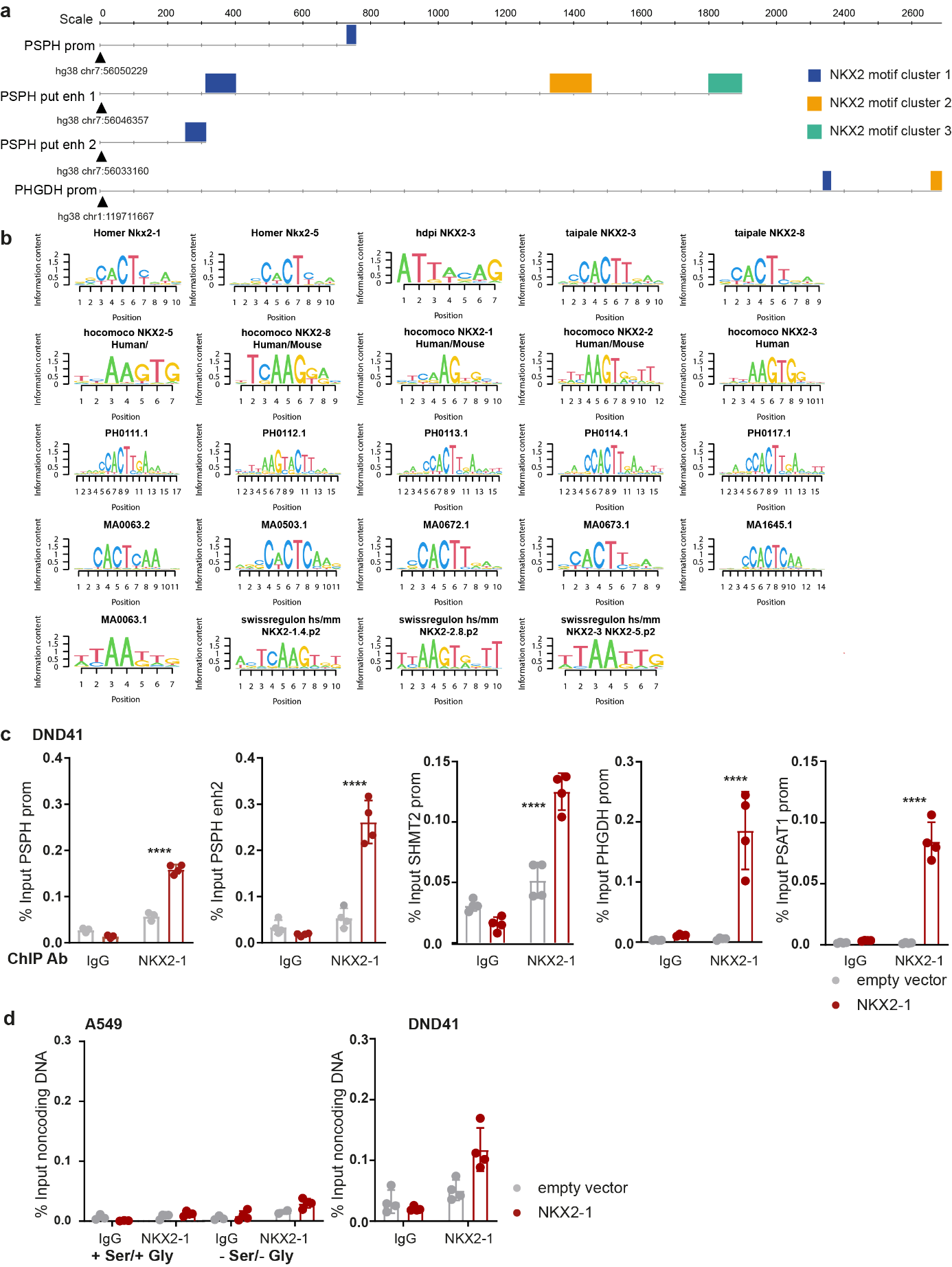
**

**Supplementary figure 2. NKX2-1 binds to regulatory regions of serine/glycine synthesis genes.**

**a)** Cluster-Buster (cBust) analysis of clustered motif occurrences of various previously described NKX motifs in the promoter and putative enhancer regions of *PSPH* and the promoter of *PHGDH.* Data were visualized with the TOUCAN tool. Motifs included in NKX2 motif clusters are defined in Supplementary table 4.

**b)** Sequence logos of the NKX DNA motifs used for the Cluster-Buster (cBust) analysis. Position weight matrices were visualized by means of the R package seqLogo (version 1.60.0).

**c)** NKX2-1 ChIP-qPCR results for the *PHGDH* promoter, *PSAT1* promoter, *PSPH* promoter and putative enhancer 2 region and *SHMT2* promoter obtained in DND41 cells cultured with serine and glycine (n=4 technical replicates).

**d)** NKX2-1 ChIP-qPCR results for a non-coding DNA region obtained in either A549 cells cultured with or without serine and glycine or DND41 cells cultured with serine and glycine (n=4 technical replicates).

Data are represented as mean ± standard deviation. Individual dots represent independent observations. Statistical analysis *p-value < 0.05, **p-value < 0.01, ***p-value < 0.001, ****p-value <0.0001. p-values were calculated using a two-tailed Student’s t-test.


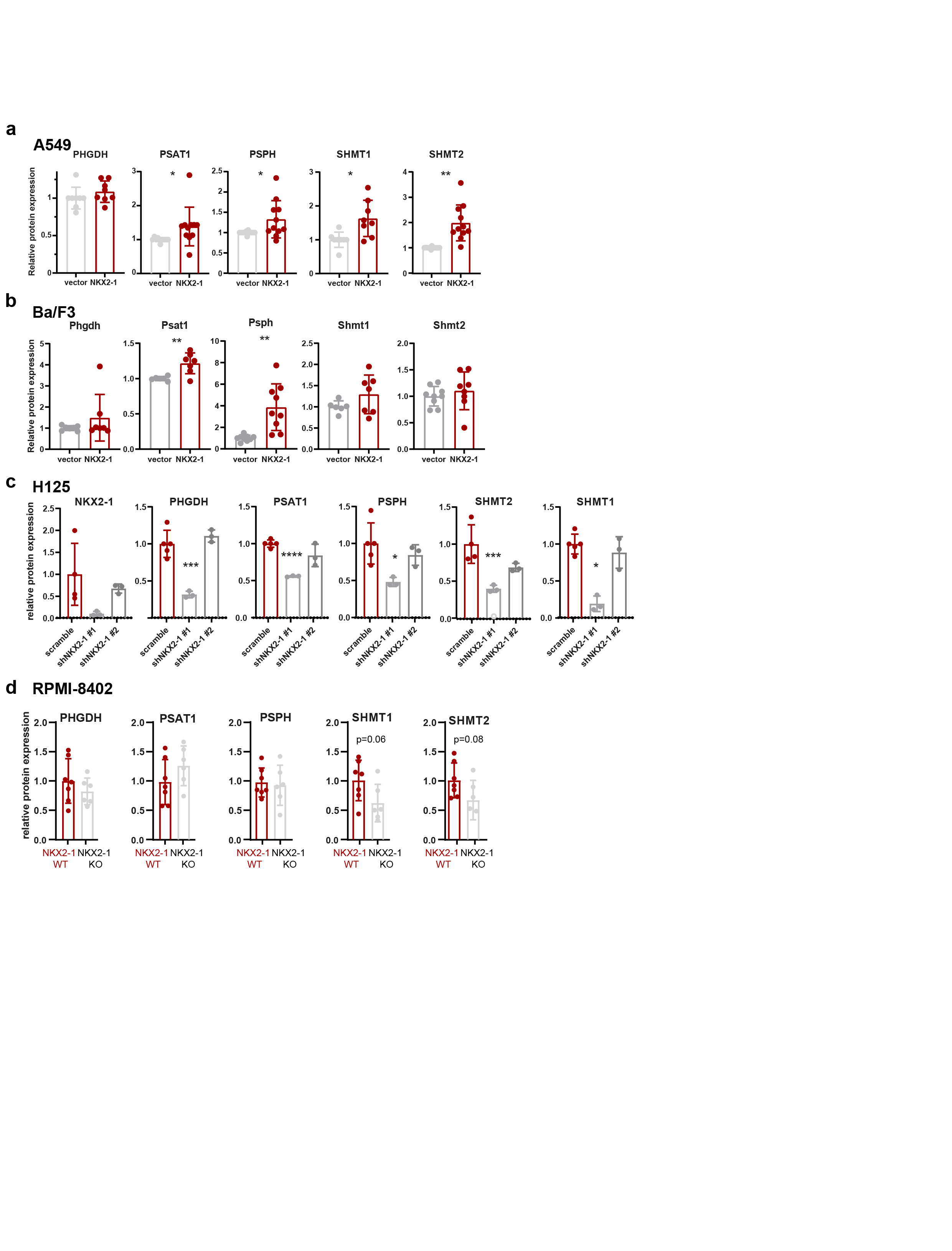


**Supplementary figure 3.** **Quantification of serine/glycine synthesis enzyme protein expression levels.**

**a-b)** Relative protein expression levels of PHGDH, PSAT1, PSPH, SHMT1 and SHMT2 in empty vector control versus NKX2-1 overexpressing A549 cells (n≥8 biological replicates) **(a)**, and Ba/F3 cells (n≥7 biological replicates) **(b)**.

**c)** Relative protein expression levels of NKX2-1, PHGDH, PSAT1, PSPH, SHMT1 and SHMT2 in scramble control versus *NKX2-1* knockdown NCI-H125 cells (n≥3 biological replicates). Only samples with at least 20% *NKX2-1* knockdown were analyzed.

**d)** Relative protein expression levels of PHGDH, PSAT1, PSPH, SHMT1 and SHMT2 in *NKX2-1* WT vs CRISPR-Cas9 *NKX2-1* KO RPMI-8402 cells (n=6 biological replicates)

Data are represented as mean ± standard deviation. Individual dots represent independent observations. Statistical analysis *p-value < 0.05, **p-value < 0.01, ***p-value < 0.001, ****p-value <0.0001. P-values were calculated using a two-tailed Student’s t-test. All cells were cultured in medium without serine/glycine for 4h prior to pellet collection.


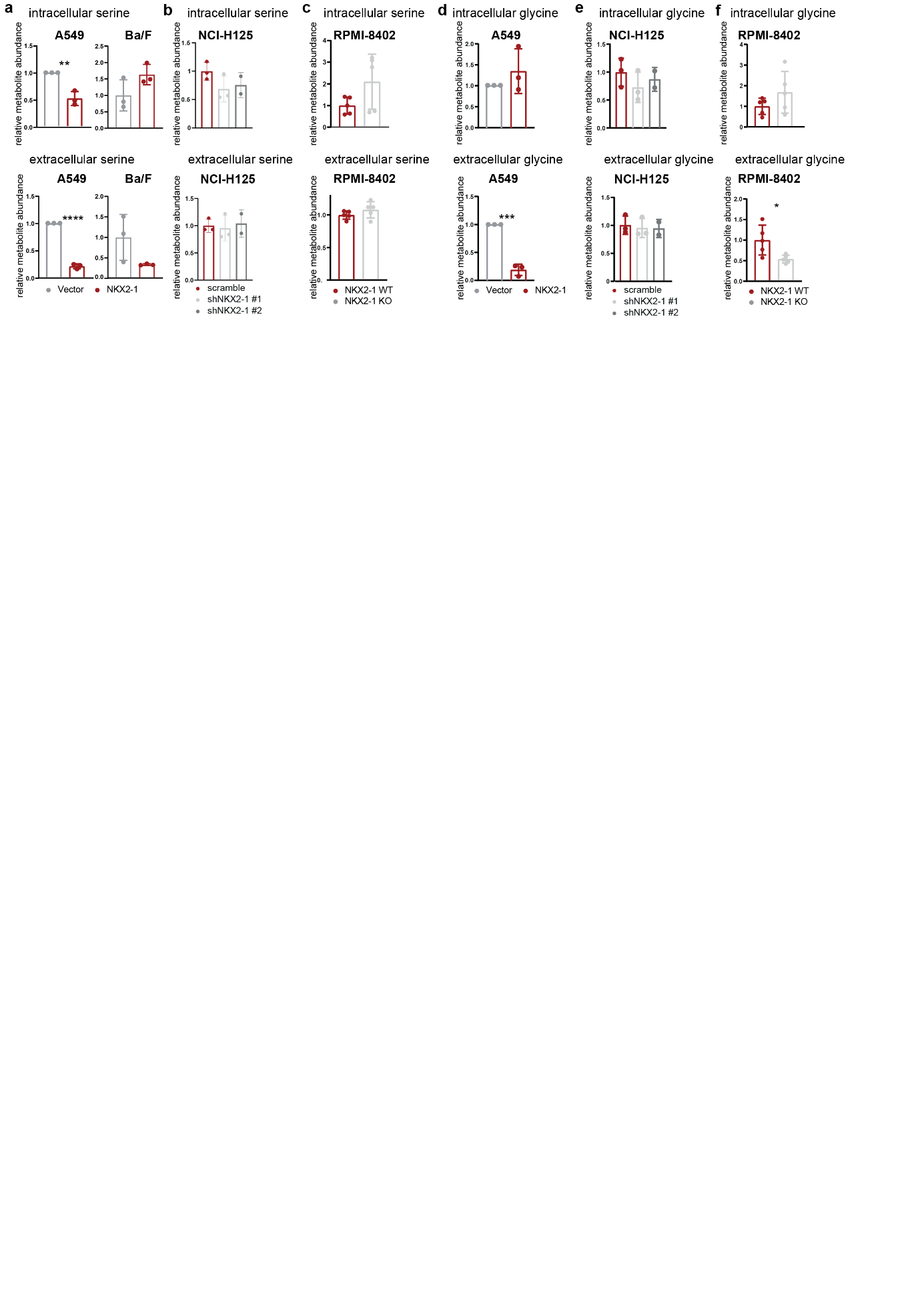


**Supplementary figure 4. NKX2-1 expression has little effects on total serine/glycine levels.**

**a)** Relative abundance of intracellular and extracellular serine in empty vector control versus NKX2-1 overexpressing A549 and Ba/F3 cells (n=3).

**b)** Relative abundance of intracellular and extracellular serine in scramble control versus *NKX2-1* knockdown NCI-H125 (n=3 for scramble and shNKX2-1 #1; n=2 for shNKX2-1 #2).

**c)** Relative abundance of intracellular and extracellular serine in *NKX2-1* WT vs CRISPR-Cas9 *NKX2-1* KO RPMI-8402 cells (n=5).

**d)** Relative abundance of intracellular and extracellular glycine in empty vector control versus NKX2-1 overexpressing A549 cells (n=3).

**e)** Relative abundance of intracellular and extracellular glycine in scramble control versus *NKX2-1* knockdown NCI-H125 (n=3 for scramble and shNKX2-1 #1; n=2 for shNKX2-1 #2).

**f)** Relative abundance of intracellular and extracellular glycine in *NKX2-1* WT vs CRISPR-Cas9 *NKX2-1* KO RPMI-8402 cells (n=5).

Data are represented as mean ± standard deviation. Individual dots represent independent observations. Statistical analysis *p-value < 0.05, **p-value < 0.01, ***p-value < 0.001, ****p-value <0.0001. P-values were calculated using a two-tailed Student’s t-test. All cells were cultured in medium without serine/glycine.


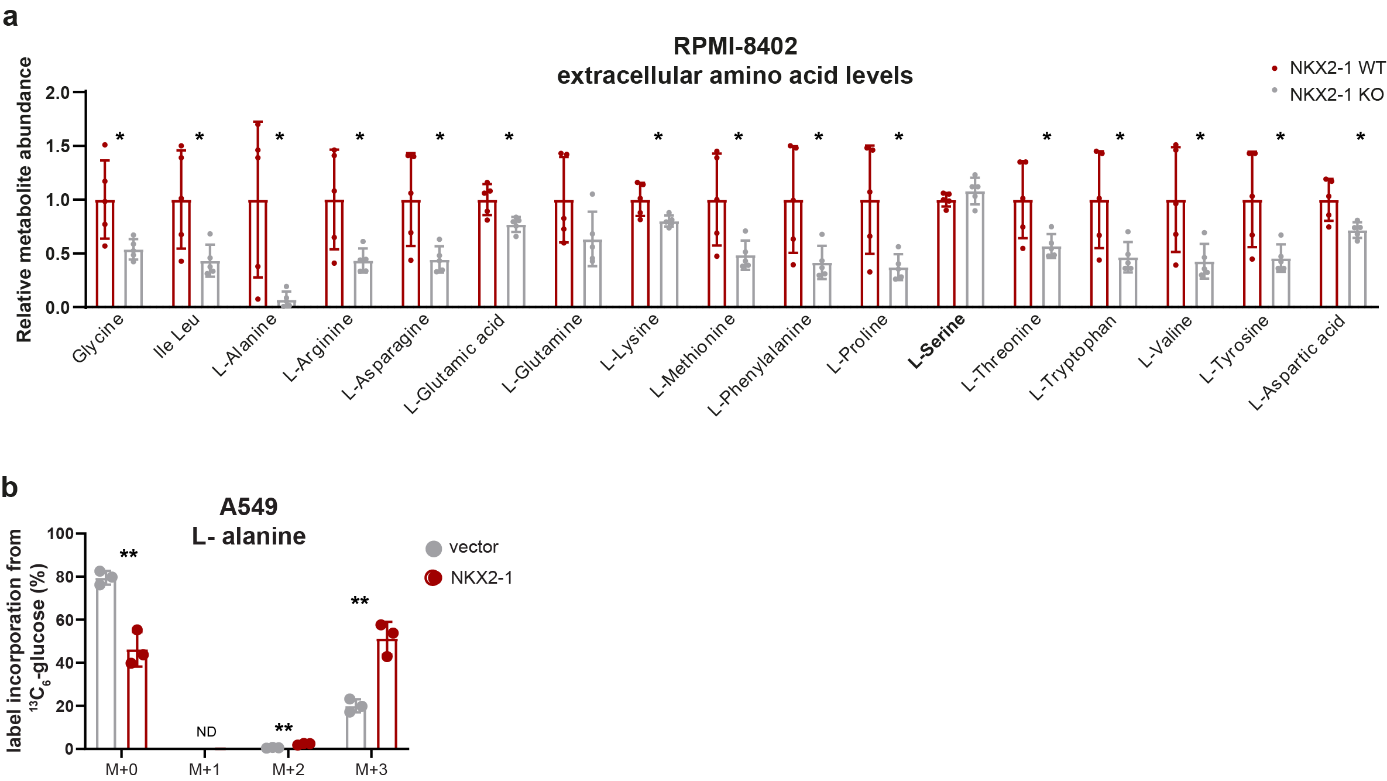


**Supplementary figure 5. NKX2-1 induced changes in amino acid metabolism.**

**a)** Relative abundance of extracellular amino acid levels in *NKX2-1* WT vs CRISPR-Cas9 *NKX2-1* KO RPMI-8402 cells (n=5).

**b)** Metabolic tracer analysis using ^13^C_6_-glucose, measuring labeled L-alanine in empty vector control versus NKX2-1 overexpressing A549 cells (n=3).

Data are represented as mean ± standard deviation. Individual dots represent independent observations. Statistical analysis *p-value < 0.05, **p-value < 0.01, ***p-value < 0.001, ****p-value <0.0001. P-values were calculated using a two-tailed Student’s t-test. Cells were cultured in medium without serine/glycine.


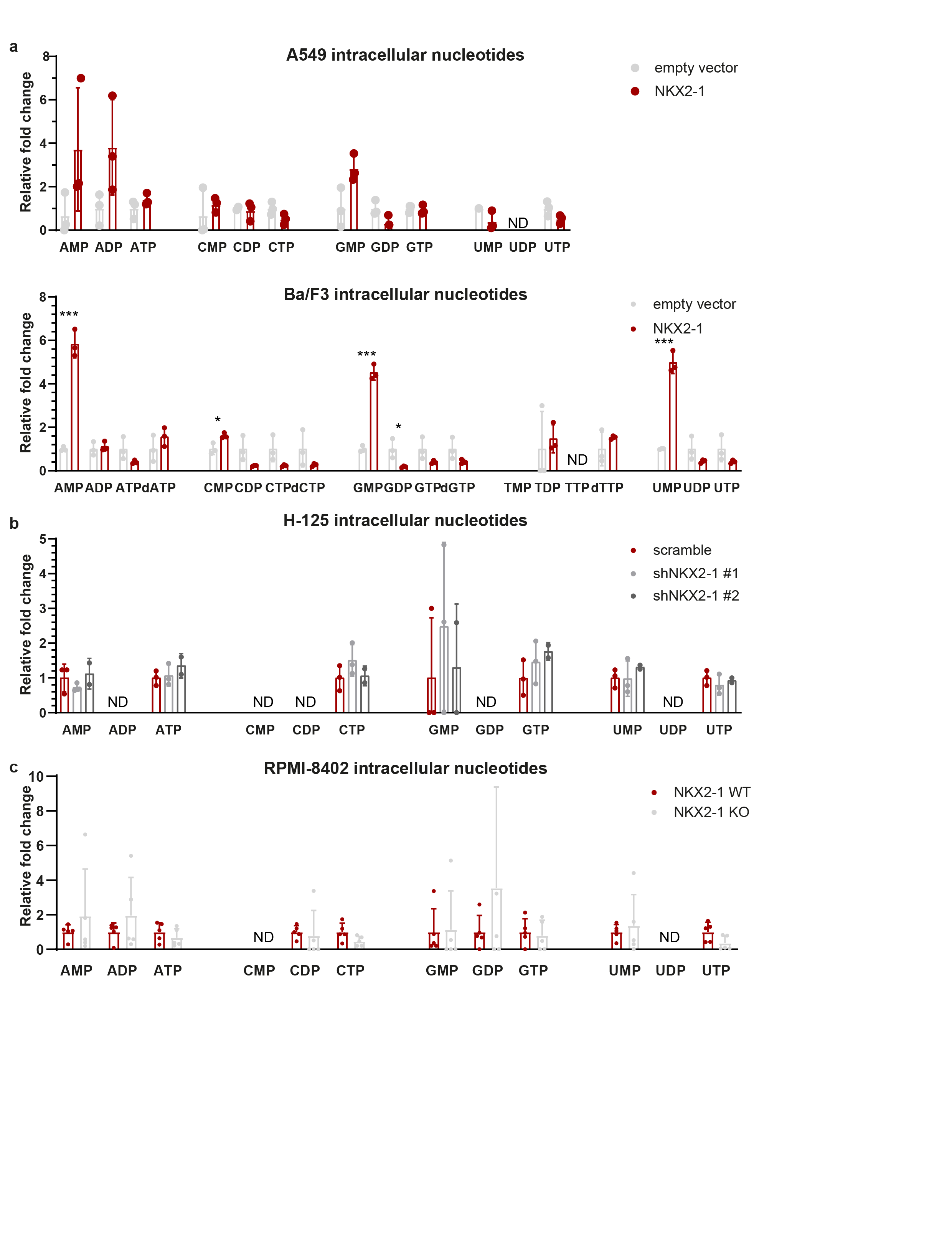


**Supplementary figure 6. Intracellular nucleotide abundances in the different cell lines.**

**a-c)** Relative abundance of intracellular nucleotides in empty vector control versus NKX2-1 overexpressing A549 cells (n=3) and Ba/F3 cells (n=3) (**a**) and in scramble control versus *NKX2-1* knockdown NCI-H125 (n=3 for scramble and shNKX2-1 #1; n=2 for shNKX2-1 #2) (**b**) and in *NKX2-1* WT vs CRISPR-Cas9 *NKX2-1* KO RPMI-8402 cells (n=5) (**c**).

Data are represented as mean ± standard deviation. Individual dots represent independent observations. Statistical analysis *p-value < 0.05, **p-value < 0.01, ***p-value < 0.001, ****p-value <0.0001. P-values were calculated using a two-tailed Student’s t-test. All cells were cultured in medium without serine/glycine.


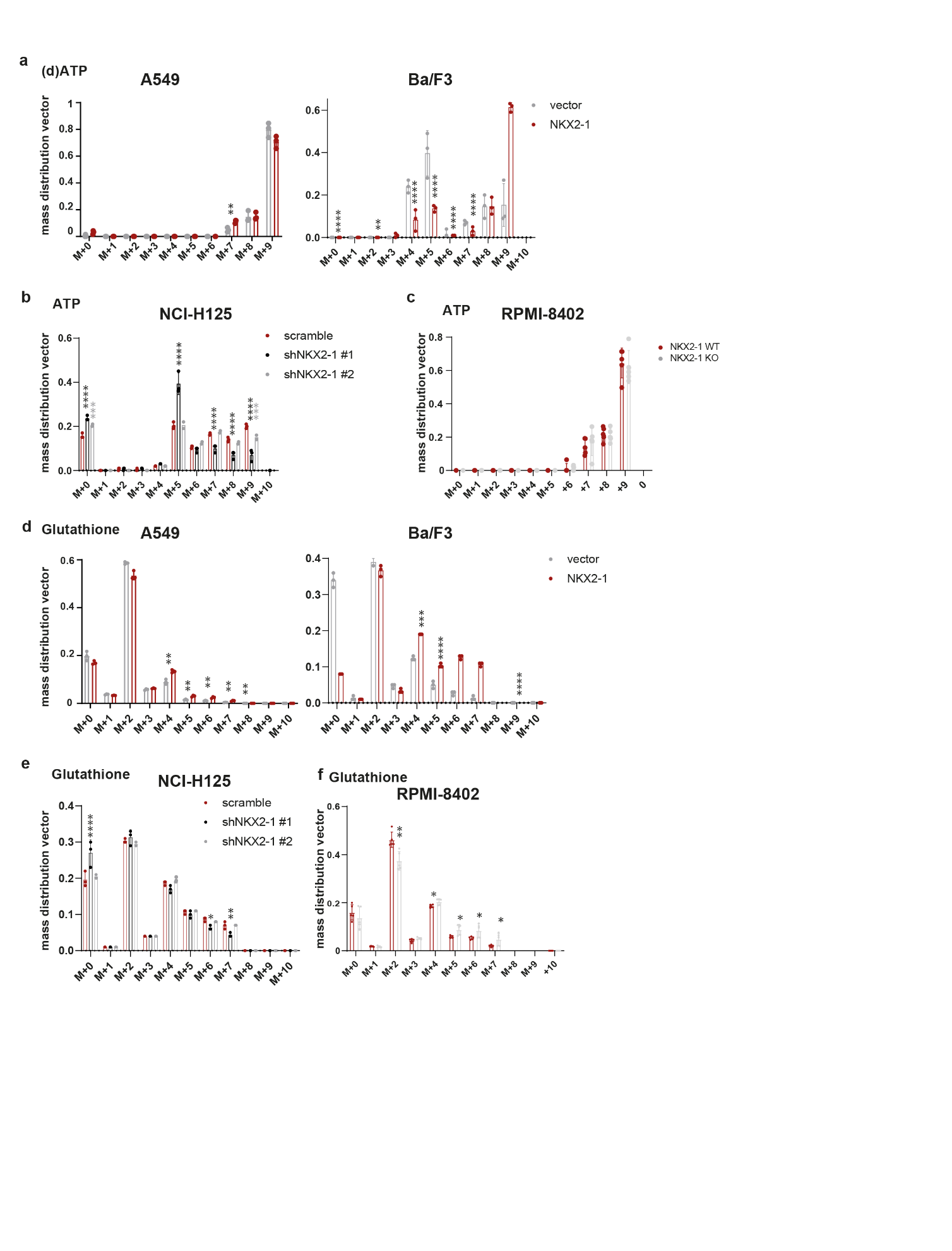


**Supplementary figure 7. NKX2-1 expression induces extensive metabolic changes in the mass distribution vector of ATP and GSH.**

**a)** (d)ATP mass distribution in empty vector control versus NKX2-1 overexpressing A549 (ATP) and Ba/F3 (dATP) cells (n=3).

**b)** ATP mass distribution in scramble control versus *NKX2-1* knockdown NCI-H125 (n=3 for scramble and shNKX2-1 #1; n=2 for shNKX2-1 #2).

**c)** ATP mass distribution in *NKX2-1* WT vs CRISPR-Cas9 *NKX2-1* KO RPMI-8402 cells (n=5).

**d)** Glutathione mass distribution in empty vector control versus NKX2-1 overexpressing A549 and Ba/F3 cells (n=3).

**e)** Glutathione mass distribution in scramble control versus *NKX2-1* knockdown NCI-H125 (n=3 for scramble and shNKX2-1 #1; n=2 for shNKX2-1 #2).

**f)** Glutathione mass distribution in *NKX2-1* WT versus CRISPR-Cas9 *NKX2-1* KO RPMI-8402 cells (n=5).

Data are represented as mean ± standard deviation. Individual dots represent independent observations. Statistical analysis *p-value < 0.05, **p-value < 0.01, ***p-value < 0.001, ****p-value <0.0001. P-values were calculated using a two-tailed Student’s t-test. All cells were cultured in medium without serine/glycine.


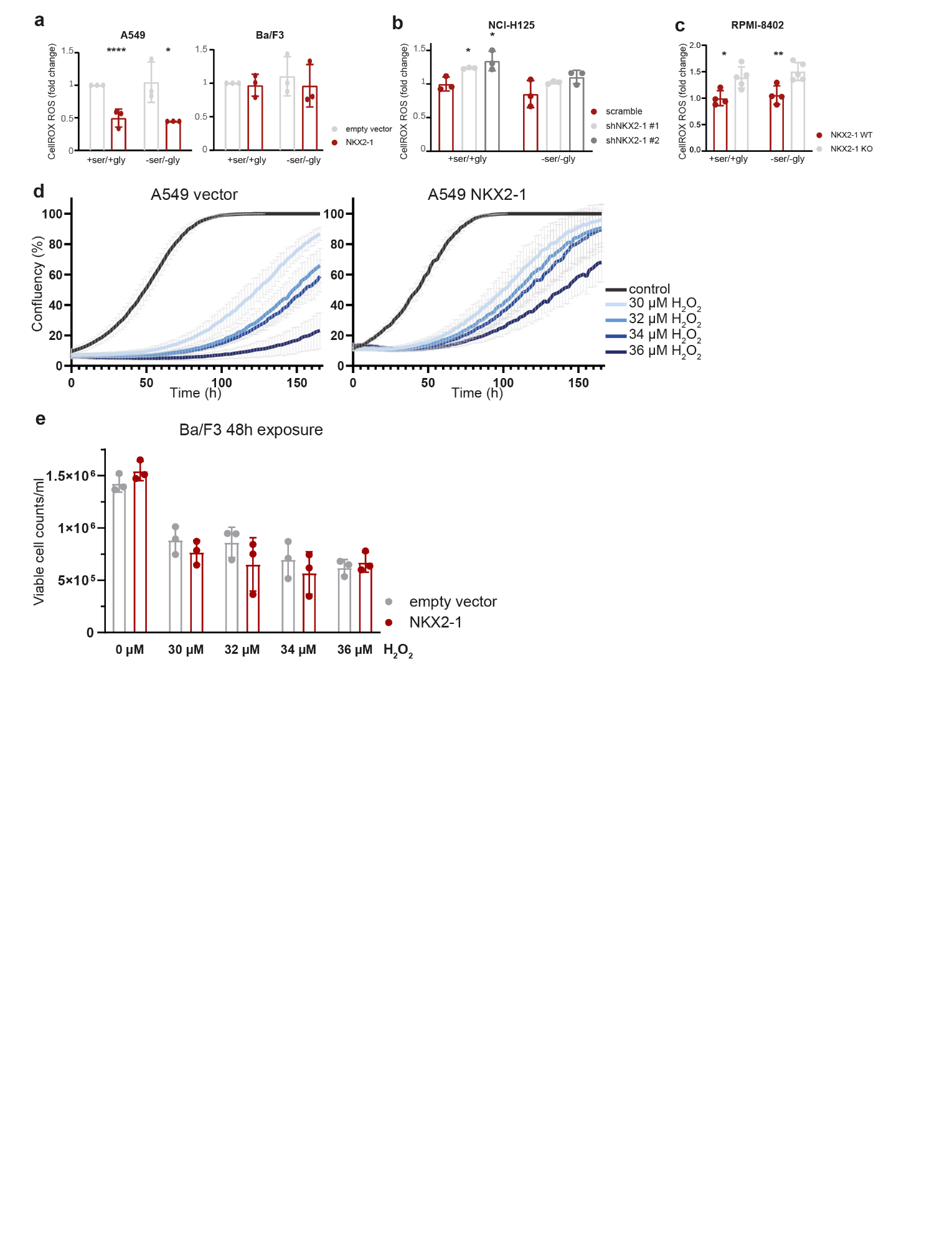


**Supplementary figure 8. NKX2-1 expression protects lung adenocarcinoma cells against oxidative stress.**

**a-c)** Flow cytometry analysis of ROS levels presenting the relative mean fluorescent intensity (MFI) of CellROX ROS staining in empty vector control versus NKX2-1 overexpressing A549 cells (n=3) and in Ba/F3 cells (n=3) (**a**) and in scramble control versus *NKX2-1* knockdown NCI-H125 cells (n=3) (**b**) and in *NKX2-1* WT vs CRISPR-cas9 *NKX2-1* KO RPMI-8402 cells (n≥4) (**c**) in medium with or without serine and glycine.

**d)** Cell confluency over time of A549 cells in the presence of increasing H_2_O_2_ concentrations in control DMEM. A representative experiment with 3 technical replicates is shown.

**e)** Viable Ba/F3 cell counts/mL after 48h of culturing in control RPMI-1640 with increasing H_2_O_2_ concentrations. Viability was determined by annexin V-PE/Zombie aqua flow cytometry (n=3).

Data are represented as mean ± standard deviation. Individual dots represent independent observations. Statistical analysis *p-value < 0.05, **p-value < 0.01, ***p-value < 0.001, ****p-value <0.0001. P-values were calculated using a two-tailed Student’s t-test.


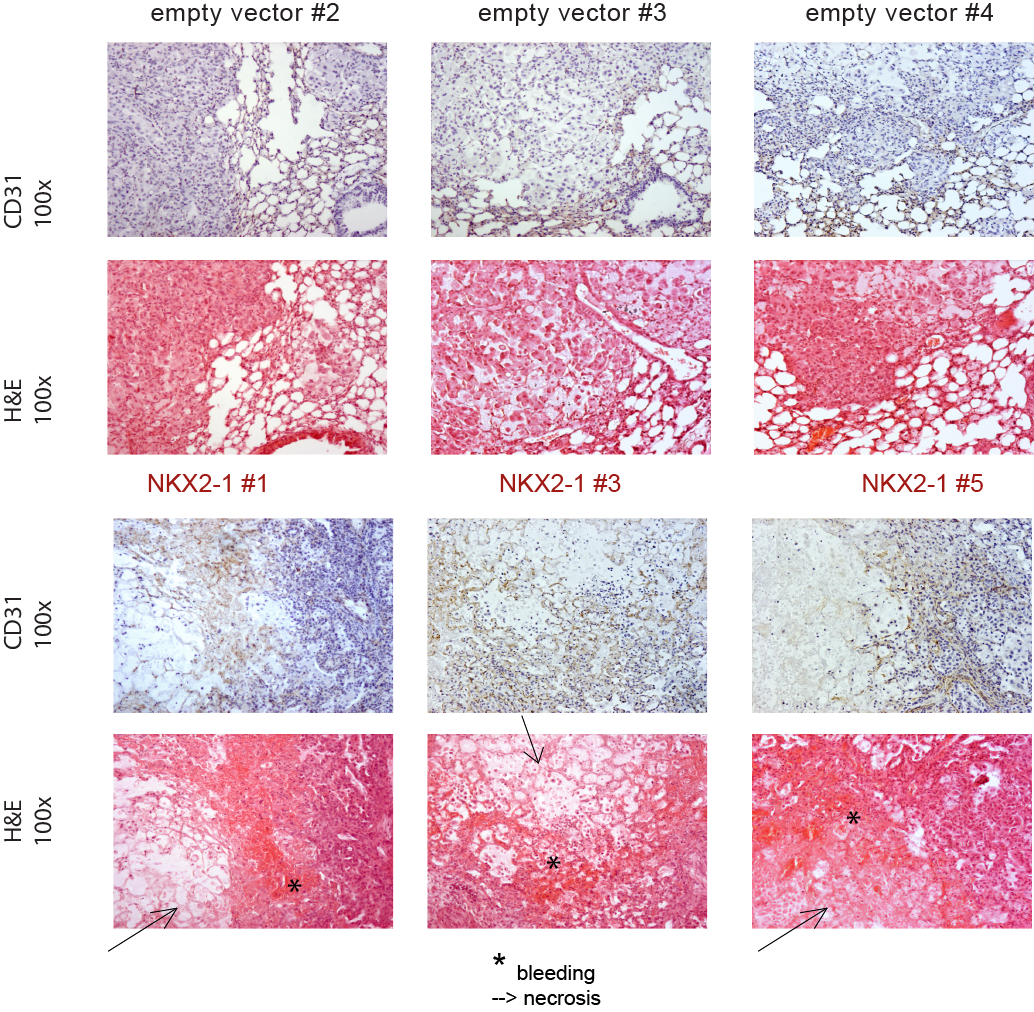


**Supplementary figure 9. CD31 staining of lung sections**.

Representative images of H&E and CD31 immunohistochemical stainings in lung sections of mice injected with empty vector control vs NKX2-1 overexpressing A549 cells at 11 weeks after cancer cell injection. Bleedings are annotated with asterisks and necrosis with arrows.

**
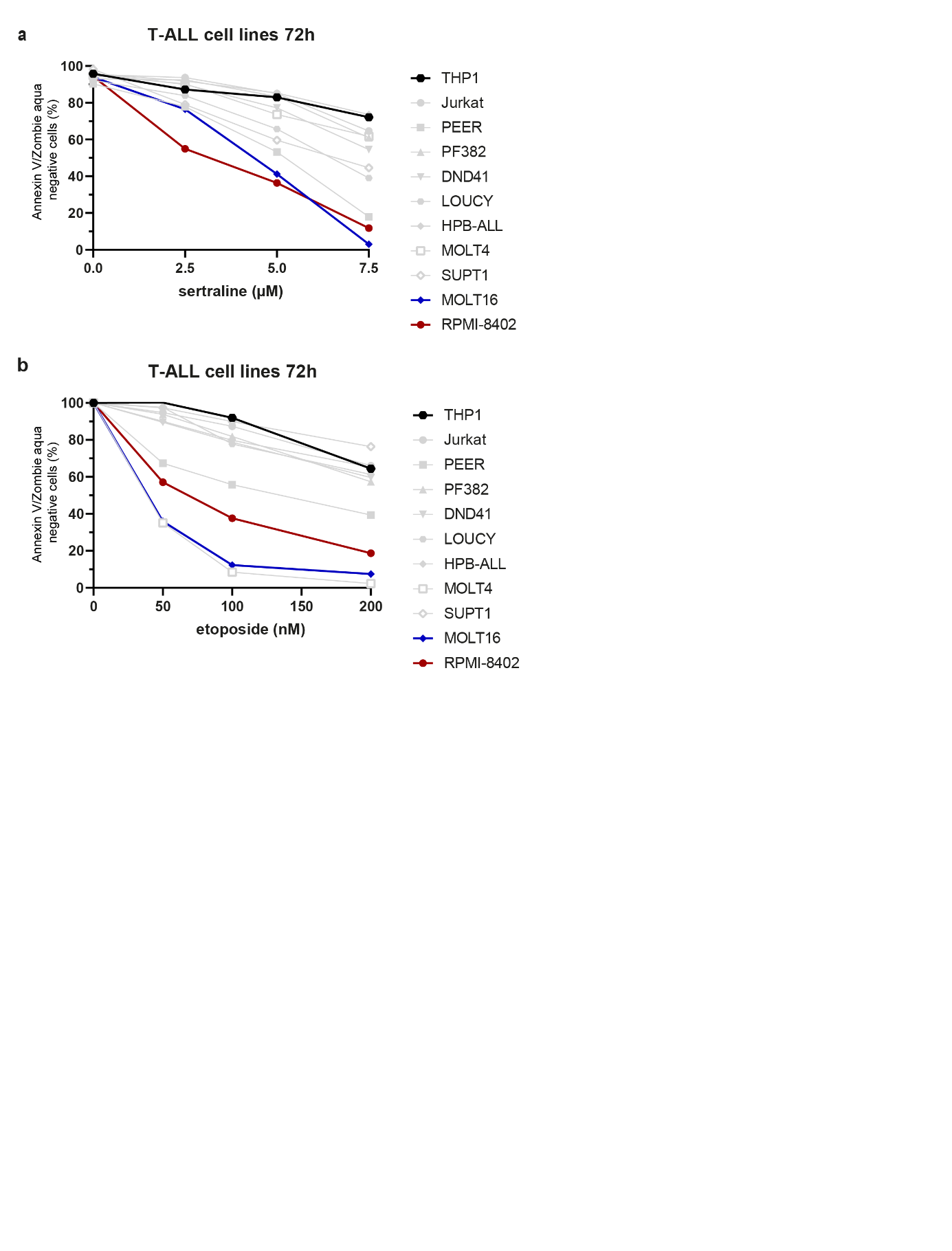
**

**Supplementary figure 10. Viability of T-ALL cells treated with sertraline and etoposide.**

**a)** Viability as determined by flow cytometry analysis of annexin V-PE/Zombie aqua staining of T-ALL cells after 72h of culturing with increasing concentrations of sertraline in RPMI-1640 containing serine and glycine (n≥3).

**b)** Viability as determined by flow cytometry analysis of annexin V-PE/Zombie aqua staining of T-ALL cells after 72h of culturing with increasing concentrations of etoposide in RPMI-1640 containing serine and glycine (n=7/8).

Data are represented as mean ± standard deviation. Individual dots represent independent observations. Statistical analysis *p-value < 0.05, **p-value < 0.01, ***p-value < 0.001, ****p-value <0.0001. P-values were calculated using a two-tailed Student’s t-test.


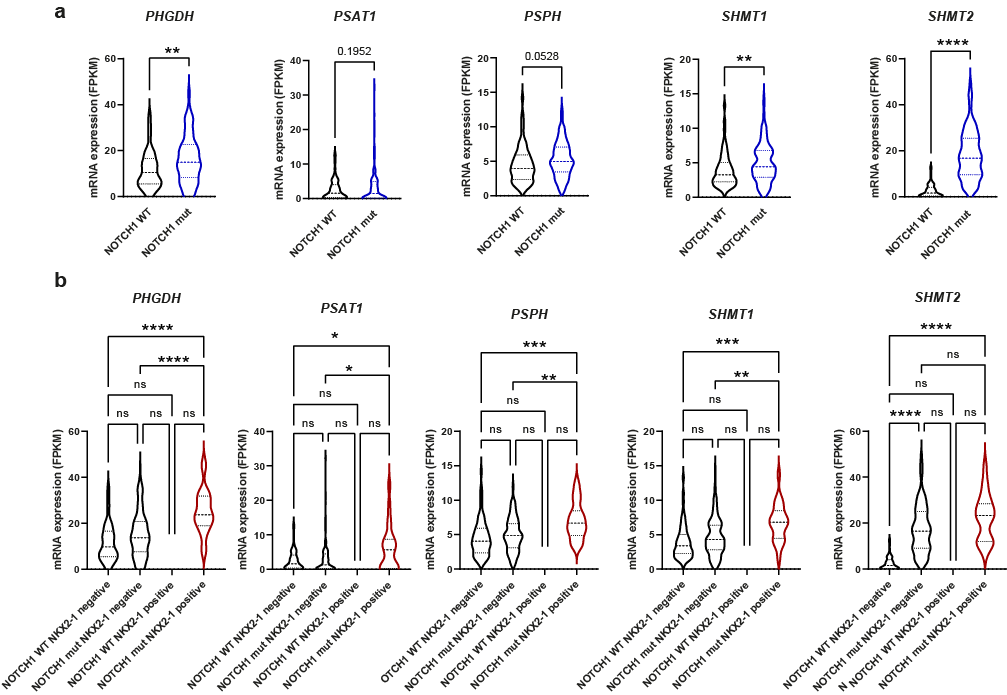


**Supplementary Figure 11. Link between NOTCH1 mutations and mRNA expression levels of serine/glycine synthesis enzymes.**

**a)** mRNA expression levels of the serine/glycine synthesis enzymes according to *NOTCH1* mutational state in 264 T-ALL patients^1^ (Student’s t-test).

**b)** mRNA expression levels of the serine/glycine synthesis enzymes according to *NOTCH1* and *NKX2-1* mutational state in 264 T-ALL patients^1^ (Turkeys multiple comparisons test).

All violin plots show the median and quartiles. Statistical analysis *p-value < 0.05, **p-value < 0.01, ***p-value < 0.001.

**
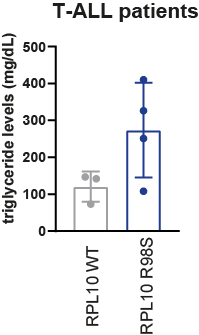
**

**Supplementary Figure 12. Triglyceride levels in T-ALL patients.**

Triglyceride blood plasma levels measured in RPL10 WT and RPL10 R98S mutated T-ALL patient samples at diagnosis (n=3 RPL10 WT and n=4 RPL10 R98S).

**Supplementary Tables**

Supplementary table 1: vector inserts

| Codon-optimized NKX2-1 | GATCTAGCCGCCGCCGAATCATGTCCATGAGTCCAAAGCACACGACACCTTTCAGCGTTTCAGATATTCTGTCCCCTCTGGAAGAGTCATATAAGAAAGTTGGCATGGAAGGTGGTGGGCTTGGAGCACCGCTGGCTGCGTACCGGCAAGGCCAGGCGGCGCCACCGACCGCAGCCATGCAGCAACATGCAGTTGGGCACCATGGCGCCGTCACAGCAGCATATCACATGACTGCCGCCGGAGTTCCTCAGTTGTCCCACTCAGCCGTGGGGGGATATTGTAACGGAAACCTTGGCAACATGTCCGAGTTGCCACCATATCAGGACACCATGCGGAATAGTGCATCAGGACCAGGTTGGTACGGAGCTAACCCTGACCCACGCTTCCCTGCGATCTCCCGCTTTATGGGACCGGCGAGCGGAATGAACATGTCCGGCATGGGTGGCTTGGGTAGTCTGGGTGACGTAAGTAAGAACATGGCGCCCCTTCCCTCCGCGCCCCGAAGAAAGAGGCGGGTTCTTTTTAGCCAAGCACAAGTGTACGAATTGGAAAGGCGATTTAAGCAGCAAAAGTACTTGTCCGCCCCTGAGAGGGAACATCTTGCGTCAATGATCCACCTGACCCCCACCCAGGTCAAAATCTGGTTTCAGAACCATCGGTATAAAATGAAGAGACAGGCGAAGGATAAAGCGGCTCAACAGCAGCTCCAGCAGGACAGTGGTGGTGGAGGAGGGGGCGGAGGAACCGGCTGTCCGCAACAGCAACAGGCTCAGCAACAATCTCCAAGGCGGGTGGCAGTCCCTGTACTTGTAAAGGATGGTAAACCCTGCCAGGCAGGTGCGCCTGCTCCAGGGGCGGCGAGTCTCCAAGGCCATGCACAGCAGCAGGCTCAACACCAAGCGCAGGCAGCCCAGGCAGCCGCAGCAGCAATAAGCGTAGGGTCCGGTGGAGCGGGTCTGGGTGCACACCCGGGGCACCAACCTGGGTCTGCAGGGCAATCTCCCGATCTTGCCCACCACGCGGCCTCCCCGGCCGCGCTTCAAGGCCAGGTATCAAGCCTGAGCCACCTGAATTCTTCCGGTTCTGACTATGGCACCATGTCCTGTTCTACCCTGCTCTATGGCCGCACCTGGTGAGAGGACGCCGGGCCGCTCG |
| --- | --- |
| shNKX2-1 #1 | ACCAGGACACCATGAGGAA |
| shNKX2-1 #2 | CGCTTGTAAATACCAGGATTT |

Supplementary table 2: primer sequences

| *ACTB* forward | GTCACCAACTGGGACGACAT |
| --- | --- |
| *ATCB* reverse | GAGGCGTACAGGGATAGCAC |
| Endogenous *NKX2-1* forward | AACCAAGCGCATCCAATCTCAAGG |
| Endogenous *NKX2-1* reverse | TGTGCCCAGAGTGAAGTTTGGTCT |
| Vector *NKX2-1* forward | AGGGCAATCTCCCGATCTT |
| Vector *NKX2-1* reverse | GGAAGAATTCAGGTGGCTCA |
| *PHGDH* forward | GCAAATCTGCGGAAAGTGCT |
| *PHGDH* reverse | TGGAGTTTCTCAGCTGCGTT |
| *PSAT1* forward | AAAAACAATGGAGGTGCCGC |
| *PSAT1* reverse | GGCTCCACTGGACAAACGTA |
| *PSPH* forward | TGTCAGAAATGACACGGCGA |
| *PSPH* reverse | GGGGGTTGCTCTGCTATGAG |
| *SHMT1* forward | CTTCCTGAGCTCGAGCGGT |
| *SHMT1* reverse | CATCGTCATTGCACTGGTTCG |
| *SHMT2* forward | CTCTTTGTTTTGGGCGGCTC |
| *SHMT2* reverse | GACACTGCCTGTCCTTCTCC |
| *Actb* forward | CATTGCTGACAGGATGCAGAAGG |
| *Atcb* reverse | TGCTGGAAGGTGGACAGTGAGG |
| *Phgdh* forward | GATGCTCAACGGAGCTGTCT |
| *Phgdh* reverse | AGGCCAATCATAGTGGGCAG |
| *Psat1* forward | AAGCTGCCACACTCGGTATT |
| *Psat1* reverse | GCCAGACCCACCTCCTTGTA |
| *Psph* forward | G​A​G​A​T​G​G​A​G​C​T​A​C​G​G​A​C​A​T​G​G​A​A​G |
| *Psph* reverse | C​T​C​C​T​C​C​A​G​T​T​C​T​C​C​C​A​G​C​A​G​C​T​C |
| *Shmt1* forward | GAGAATTTTGCCAGCCGAGC |
| *Shmt1* reverse | GCCTGTAATGCACGCTTCTG |
| *Shmt2* forward | ACTCACAAGACACTGCGAGG |
| *Shmt2* reverse | GTACTCGCGGAACATAGGGG |
| *PSPH* putative enhancer 2 forward (ChIP-qPCR) | TCCACCGAACGCTCTCTTG |
| *PSPH* putative enhancer 2 reverse (ChIP-qPCR) | GCTTTGCAGAGCAGACAGAT |
| *PSPH* promoter forward (ChIP-qPCR) | CTTGTCGTCATCGCGCAC |
| *PSPH* promoter reverse (ChIP-qPCR) | CCCAAAGAGCTCCAACGCC |
| *SHMT2* promoter forward (ChIP-qPCR) | GGTGAGTGGGCGAACTACAA |
| *SHMT2* promoter reverse (ChIP-qPCR) | TAACGGGAGGGATCTACGCA |
| *PHGDH* promoter forward (ChIP-qPCR) | \| CGCGGGAGGATAATAAAGCG \| \| --- \| |
| *PHGDH* promoter reverse (ChIP-qPCR) | ACTCAAACTCTCCGCGACTC |
| *PSAT1* promoter forward (ChIP-qPCR) | CGCGAGGAGGAGCAACTG |
| *PSAT1* promoter reverse (ChIP-qPCR) | \| AGGAGCTCACATCCCCATTG \| \| --- \| |
| Non-coding DNA forward (ChIP-qPCR) | ATCCGTTGCTATTCCCGGTC |
| Non-coding DNA reverse (ChIP-qPCR) | AGAGGAAGACCAATCTGCGG |

Supplementary table 3: Antibodies

| **Antibody** | **Company** | **Product number** | **Dilution** |
| --- | --- | --- | --- |
| PHGDH | Protein Tech | 14719-1-AP | 1:1000 |
| PSAT1 | Protein Tech | 10501-1-AP | 1:1000 |
| PSPH | Protein Tech | 14513-1-AP | 1:1000 |
| SHMT1 | Abcam | ab186130 | 1:1000 |
| SHMT2 | Protein Tech | 1099-1-AP | 1:1000 |
| TTF-1 | Abcam | ab76013 | 1:1000 |
| VINCULIN | Sigma | V9131 | 1:25000 |
| Β-ACTIN | Sigma | A1978 | 1:25000 |
| Goat anti-Rabbit IgG-HRP | ThermoFisher Scientific | 31462 | 1:5000 |
| Goat anti-Mouse IgG-HRP | ThermoFisher Scientific | 31432 | 1:5000 |
| CD31 | Cell signaling | 77699 | 1:100 |

Supplementary table 4: NKX2 motif clusters

| **Cluster** | **Location** | **Motif ID** |
| --- | --- | --- |
| NKX2 motif cluster 1 | PSPH promoter | Hocomoco NKX2-1 Human/Mouse |
| NKX2 motif cluster 1 | PSPH promoter | Hocomoco NKX2-2 Human/Mouse |
| NKX2 motif cluster 1 | PSPH promoter | Hocomoco NKX2-3 Human |
| NKX2 motif cluster 1 | PSPH promoter | Hocomoco NKX2-5 Mouse |
| NKX2 motif cluster 1 | PSPH promoter | Hocomoco NKX2-8 Human |
| NKX2 motif cluster 1 | PSPH promoter | Homer Nkx2-5 |
| NKX2 motif cluster 1 | PSPH promoter | Homer Nkx2-1 |
| NKX2 motif cluster 1 | PSPH promoter | MA0063.1 |
| NKX2 motif cluster 1 | PSPH promoter | MA0063.2 |
| NKX2 motif cluster 1 | PSPH promoter | MA0672.1 |
| NKX2 motif cluster 1 | PSPH promoter | MA0673.1 |
| NKX2 motif cluster 1 | PSPH promoter | Swissregulon hs/mm NKX2-1.4.p2 |
| NKX2 motif cluster 1 | PSPH promoter | Swissregulon hs/mm NKX2-3 NKX2-5.p2. |
| NKX2 motif cluster 1 | PSPH promoter | Taipale NKX2-3 |
| NKX2 motif cluster 1 | PSPH promoter | Taipale NKX2-8 |
| NKX2 motif cluster 1 | PSPH putative enhancer 1 | Hocomoco NKX2-1 Human/Mouse |
| NKX2 motif cluster 1 | PSPH putative enhancer 1 | Hocomoco NKX2-2 Human/Mouse |
| NKX2 motif cluster 1 | PSPH putative enhancer 1 | Hocomoco NKX2-3 Human |
| NKX2 motif cluster 1 | PSPH putative enhancer 1 | Hocomoco NKX2-5 Human/Mouse |
| NKX2 motif cluster 1 | PSPH putative enhancer 1 | Hocomoco NKX2-8 Human/Mouse |
| NKX2 motif cluster 1 | PSPH putative enhancer 1 | Homer Nkx2-5 |
| NKX2 motif cluster 1 | PSPH putative enhancer 1 | Homer Nkx2-1 |
| NKX2 motif cluster 1 | PSPH putative enhancer 1 | MA0063.1 |
| NKX2 motif cluster 1 | PSPH putative enhancer 1 | MA0063.2 |
| NKX2 motif cluster 1 | PSPH putative enhancer 1 | MA0503.1 |
| NKX2 motif cluster 1 | PSPH putative enhancer 1 | MA0672.1 |
| NKX2 motif cluster 1 | PSPH putative enhancer 1 | MA0673.1 |
| NKX2 motif cluster 1 | PSPH putative enhancer 1 | MA1645.1 |
| NKX2 motif cluster 1 | PSPH putative enhancer 1 | PH0111.1 |
| NKX2 motif cluster 1 | PSPH putative enhancer 1 | PH0113.1 |
| NKX2 motif cluster 1 | PSPH putative enhancer 1 | PH0114.1 |
| NKX2 motif cluster 1 | PSPH putative enhancer 1 | PH0171.1 |
| NKX2 motif cluster 1 | PSPH putative enhancer 1 | Swissregulon hs/mm NKX2-1.4.p2 |
| NKX2 motif cluster 1 | PSPH putative enhancer 1 | Swissregulon hs/mm NKX2-3 NKX2-5.p2 |
| NKX2 motif cluster 1 | PSPH putative enhancer 1 | Swissregulan mm NKX2-2.8.p2 |
| NKX2 motif cluster 1 | PSPH putative enhancer 1 | Taipale NKX2-3 |
| NKX2 motif cluster 1 | PSPH putative enhancer 1 | Taipale NKX2-8 |
| NKX2 motif cluster 2 | PSPH putative enhancer 1 | hdpi NKX2-3 |
| NKX2 motif cluster 2 | PSPH putative enhancer 1 | Hocomoco NKX2-1 Human/Mouse |
| NKX2 motif cluster 2 | PSPH putative enhancer 1 | Hocomoco NKX2-2 Human/Mouse |
| NKX2 motif cluster 2 | PSPH putative enhancer 1 | Hocomoco NKX2-3 Human |
| NKX2 motif cluster 2 | PSPH putative enhancer 1 | Hocomoco NKX2-5 Human/Mouse |
| NKX2 motif cluster 2 | PSPH putative enhancer 1 | Hocomoco NKX2-8 Human/Mouse |
| NKX2 motif cluster 2 | PSPH putative enhancer 1 | Homer Nkx2-5 |
| NKX2 motif cluster 2 | PSPH putative enhancer 1 | Homer Nkx2-1 |
| NKX2 motif cluster 2 | PSPH putative enhancer 1 | MA0063.1 |
| NKX2 motif cluster 2 | PSPH putative enhancer 1 | MA0063.2 |
| NKX2 motif cluster 2 | PSPH putative enhancer 1 | MA0503.1 |
| NKX2 motif cluster 2 | PSPH putative enhancer 1 | MA0672.1 |
| NKX2 motif cluster 2 | PSPH putative enhancer 1 | MA0673.1 |
| NKX2 motif cluster 2 | PSPH putative enhancer 1 | MA1645.1 |
| NKX2 motif cluster 2 | PSPH putative enhancer 1 | PH0111.1 |
| NKX2 motif cluster 2 | PSPH putative enhancer 1 | PH0112.1 |
| NKX2 motif cluster 2 | PSPH putative enhancer 1 | PH0113.1 |
| NKX2 motif cluster 2 | PSPH putative enhancer 1 | PH0114.1 |
| NKX2 motif cluster 2 | PSPH putative enhancer 1 | PH0171.1 |
| NKX2 motif cluster 2 | PSPH putative enhancer 1 | Swissregulon hs/mm NKX2-1.4.p2 |
| NKX2 motif cluster 2 | PSPH putative enhancer 1 | Swissregulon mm NKX2-3 NKX2-5.p2 |
| NKX2 motif cluster 2 | PSPH putative enhancer 1 | Swissregulan hs/mm NKX2-2.8.p2 |
| NKX2 motif cluster 2 | PSPH putative enhancer 1 | Taipale NKX2-3 |
| NKX2 motif cluster 2 | PSPH putative enhancer 1 | Taipale NKX2-8 |
| NKX2 motif cluster 3 | PSPH putative enhancer 1 | hdpi NKX2-3 |
| NKX2 motif cluster 3 | PSPH putative enhancer 1 | Hocomoco NKX2-1 Human/Mouse |
| NKX2 motif cluster 3 | PSPH putative enhancer 1 | Hocomoco NKX2-2 Human/Mouse |
| NKX2 motif cluster 3 | PSPH putative enhancer 1 | Hocomoco NKX2-3 Human |
| NKX2 motif cluster 3 | PSPH putative enhancer 1 | Hocomoco NKX2-5 Human/Mouse |
| NKX2 motif cluster 3 | PSPH putative enhancer 1 | Hocomoco NKX2-8 Human/Mouse |
| NKX2 motif cluster 3 | PSPH putative enhancer 1 | Homer Nkx2-5 |
| NKX2 motif cluster 3 | PSPH putative enhancer 1 | Homer Nkx2-1 |
| NKX2 motif cluster 3 | PSPH putative enhancer 1 | MA0063.1 |
| NKX2 motif cluster 3 | PSPH putative enhancer 1 | MA0063.2 |
| NKX2 motif cluster 3 | PSPH putative enhancer 1 | MA0503.1 |
| NKX2 motif cluster 3 | PSPH putative enhancer 1 | MA0672.1 |
| NKX2 motif cluster 3 | PSPH putative enhancer 1 | MA0673.1 |
| NKX2 motif cluster 3 | PSPH putative enhancer 1 | MA1645.1 |
| NKX2 motif cluster 3 | PSPH putative enhancer 1 | PH0111.1 |
| NKX2 motif cluster 3 | PSPH putative enhancer 1 | PH0113.1 |
| NKX2 motif cluster 3 | PSPH putative enhancer 1 | PH0114.1 |
| NKX2 motif cluster 3 | PSPH putative enhancer 1 | PH0171.1 |
| NKX2 motif cluster 3 | PSPH putative enhancer 1 | Swissregulon hs/mm NKX2-1.4.p2 |
| NKX2 motif cluster 3 | PSPH putative enhancer 1 | Swissregulon hs/mm NKX2-3 NKX2-5.p2 |
| NKX2 motif cluster 3 | PSPH putative enhancer 1 | Swissregulan hs/mm NKX2-2.8.p2 |
| NKX2 motif cluster 3 | PSPH putative enhancer 1 | Taipale NKX2-3 |
| NKX2 motif cluster 3 | PSPH putative enhancer 1 | Taipale NKX2-8 |
| NKX2 motif cluster 1 | PSPH putative enhancer 2 | Hocomoco NKX2-1 Human/Mouse |
| NKX2 motif cluster 1 | PSPH putative enhancer 2 | Hocomoco NKX2-2 Human/Mouse |
| NKX2 motif cluster 1 | PSPH putative enhancer 2 | Hocomoco NKX2-3 Human |
| NKX2 motif cluster 1 | PSPH putative enhancer 2 | Hocomoco NKX2-5 Human/Mouse |
| NKX2 motif cluster 1 | PSPH putative enhancer 2 | Hocomoco NKX2-8 Human/Mouse |
| NKX2 motif cluster 1 | PSPH putative enhancer 2 | Homer Nkx2-5 |
| NKX2 motif cluster 1 | PSPH putative enhancer 2 | Homer Nkx2-1 |
| NKX2 motif cluster 1 | PSPH putative enhancer 2 | MA0063.1 |
| NKX2 motif cluster 1 | PSPH putative enhancer 2 | MA0063.2 |
| NKX2 motif cluster 1 | PSPH putative enhancer 2 | MA0503.1 |
| NKX2 motif cluster 1 | PSPH putative enhancer 2 | MA0672.1 |
| NKX2 motif cluster 1 | PSPH putative enhancer 2 | MA0673.1 |
| NKX2 motif cluster 1 | PSPH putative enhancer 2 | MA1645.1 |
| NKX2 motif cluster 1 | PSPH putative enhancer 2 | PH0113.1 |
| NKX2 motif cluster 1 | PSPH putative enhancer 2 | PH0114.1 |
| NKX2 motif cluster 1 | PSPH putative enhancer 2 | PH0171.1 |
| NKX2 motif cluster 1 | PSPH putative enhancer 2 | Swissregulon hs/mm NKX2-1.4.p2 |
| NKX2 motif cluster 1 | PSPH putative enhancer 2 | Taipale NKX2-3 |
| NKX2 motif cluster 1 | PSPH putative enhancer 2 | Taipale NKX2-8 |
| NKX2 motif cluster 1 | PHGDH promoter | Hocomoco NKX2-1 Human/Mouse |
| NKX2 motif cluster 1 | PHGDH promoter | Hocomoco NKX2-2 Human/Mouse |
| NKX2 motif cluster 1 | PHGDH promoter | Hocomoco NKX2-3 Human |
| NKX2 motif cluster 1 | PHGDH promoter | Hocomoco NKX2-8 Human/Mouse |
| NKX2 motif cluster 1 | PHGDH promoter | Homer Nkx2-5 |
| NKX2 motif cluster 1 | PHGDH promoter | Taipale NKX2-3 |
| NKX2 motif cluster 1 | PHGDH promoter | Taipale NKX2-8 |
| NKX2 motif cluster 1 | PHGDH promoter | Hocomoco NKX2-5 Human/Mouse |
| NKX2 motif cluster 1 | PHGDH promoter | Homer Nkx2-1 |
| NKX2 motif cluster 1 | PHGDH promoter | MA0063.1 |
| NKX2 motif cluster 1 | PHGDH promoter | Swissregulon hs/mm NKX2-3 NKX2-5.p2 |
| NKX2 motif cluster 1 | PHGDH promoter | Swissregulan hs/mm NKX2-2.8.p2 |
| NKX2 motif cluster 1 | PHGDH promoter | MA1645.1 |
| NKX2 motif cluster 1 | PHGDH promoter | Hocomoco NKX2-5 Human/Mouse |
| NKX2 motif cluster 1 | PHGDH promoter | Homer Nkx2-5 |
| NKX2 motif cluster 1 | PHGDH promoter | MA0503.1 |
| NKX2 motif cluster 1 | PHGDH promoter | Homer Nkx2-1 |
| NKX2 motif cluster 1 | PHGDH promoter | MA0063.2 |
| NKX2 motif cluster 1 | PHGDH promoter | MA0673.1 |
| NKX2 motif cluster 1 | PHGDH promoter | Taipale NKX2-3 |
| NKX2 motif cluster 1 | PHGDH promoter | Taipale NKX2-8 |
| NKX2 motif cluster 1 | PHGDH promoter | MA0063.1 |
| NKX2 motif cluster 1 | PHGDH promoter | hdpi__NKX2-3 |
| NKX2 motif cluster 2 | PHGDH promoter | MA0063.1 |
| NKX2 motif cluster 2 | PHGDH promoter | Swissregulon hs/mm NKX2-3 NKX2-5.p2 |
| NKX2 motif cluster 2 | PHGDH promoter | MA0063.1 |
| NKX2 motif cluster 2 | PHGDH promoter | MA1645.1 |
| NKX2 motif cluster 2 | PHGDH promoter | Homer Nkx2-5 |
| NKX2 motif cluster 2 | PHGDH promoter | Homer Nkx2-1 |
| NKX2 motif cluster 2 | PHGDH promoter | MA0063.2 |
| NKX2 motif cluster 2 | PHGDH promoter | MA0063.1 |
| NKX2 motif cluster 2 | PHGDH promoter | MA0673.1 |
| NKX2 motif cluster 2 | PHGDH promoter | Swissregulon hs/mm NKX2-1.4.p2 |
| NKX2 motif cluster 2 | PHGDH promoter | Hocomoco NKX2-1 Human/Mouse |
| NKX2 motif cluster 2 | PHGDH promoter | Hocomoco NKX2-2 Human/Mouse |
| NKX2 motif cluster 2 | PHGDH promoter | Hocomoco NKX2-3 Human |
| NKX2 motif cluster 2 | PHGDH promoter | Hocomoco NKX2-5 Human/Mouse |
| NKX2 motif cluster 2 | PHGDH promoter | Hocomoco NKX2-8 Human/Mouse |
| NKX2 motif cluster 2 | PHGDH promoter | Taipale NKX2-3 |
| NKX2 motif cluster 2 | PHGDH promoter | Taipale NKX2-8 |

**Supplementary information**

1. ROS gating strategy


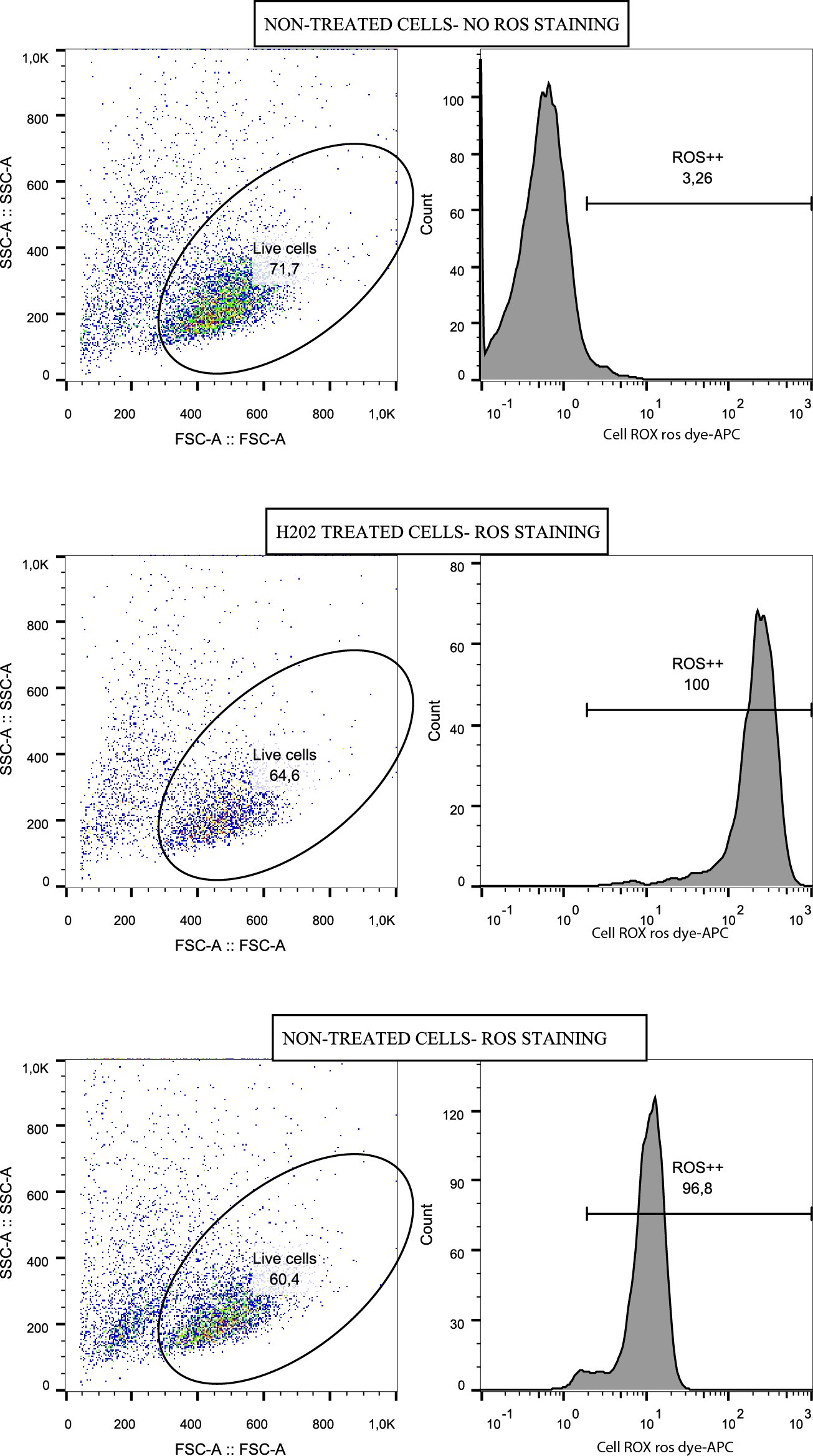


For measurement of intracellular ROS levels (Supplementary Fig.8), cells were first gated on live cells based on FSC/SSC scatter. H_2_O_2_ treated cells and baseline parental cells (without addition of ROS dye) were used as reference for gate setting of ROS positive cells. MFI of APC staining in live cells was computed afterwards.

2. Annexin-V-PE/Zombie aqua gating strategy


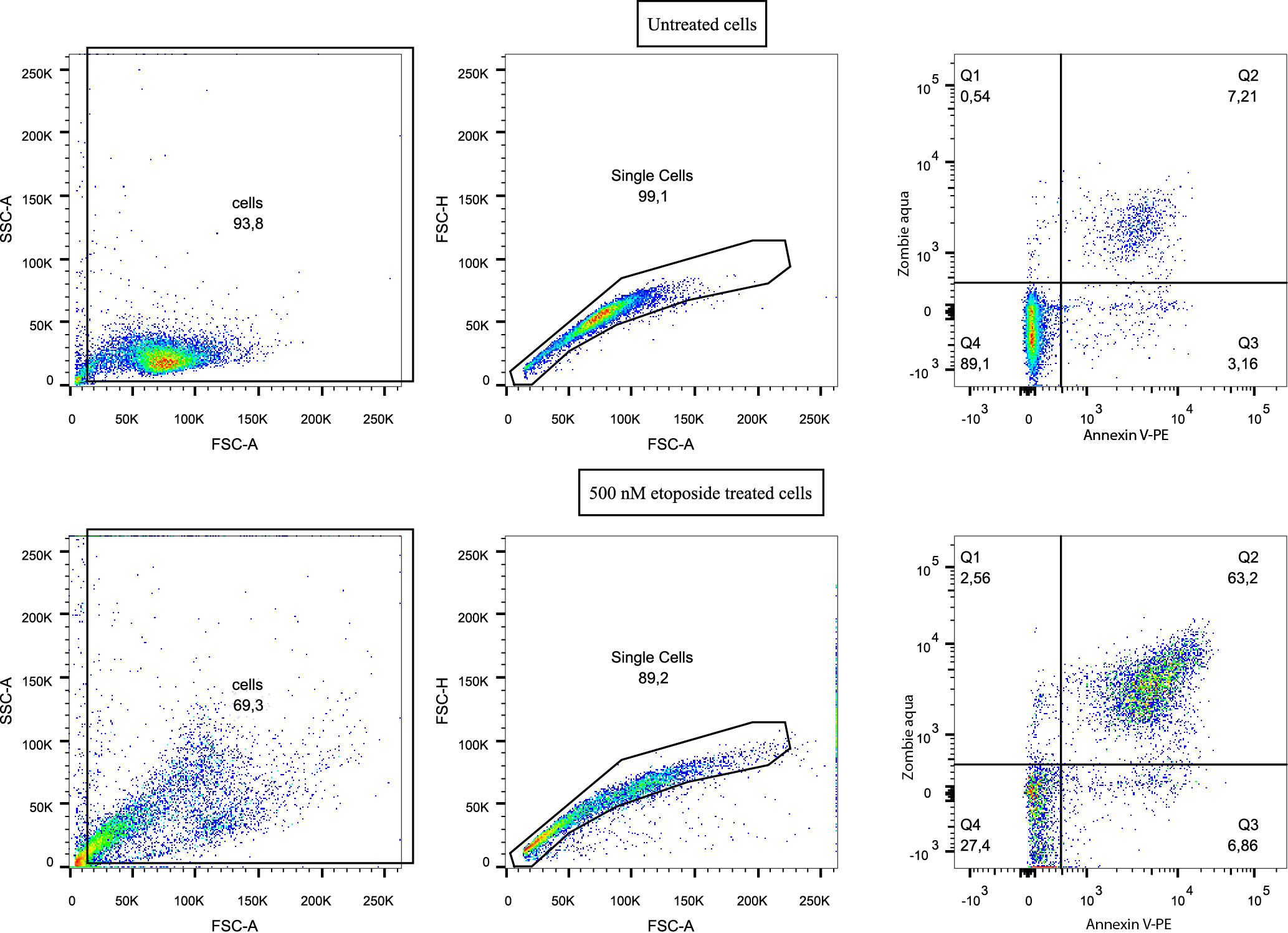


For cell viability (Annexin V-PE/Zombie aqua (measured in EFluor506 channel)), cells were first gated on FSC/SSC scatter to gate out cell debris. Next, cells were gated on FSC-A/FSC-H to select for single cells. Viable standard cultured cells were used to set the gates on this negative control, 500 nM etoposide treated cells were used to set the gates as a positive control.
